# Sex Disparities in Cardiovascular Risk Factor Assessment and Screening for Diabetes-Related Complications in Individuals With Diabetes: A Systematic Review

**DOI:** 10.3389/fendo.2021.617902

**Published:** 2021-03-30

**Authors:** Marit de Jong, Sanne A. E. Peters, Rianneke de Ritter, Carla J. H. van der Kallen, Simone J. S. Sep, Mark Woodward, Coen D. A. Stehouwer, Michiel L. Bots, Rimke C. Vos

**Affiliations:** ^1^ Julius Center for Health Sciences and Primary Care, University Medical Center Utrecht, Utrecht University, Utrecht, Netherlands; ^2^ The George Institute for Global Health, Imperial College London, London, United Kingdom; ^3^ The George Institute for Global Health, University of New South Wales, Sydney, NSW, Australia; ^4^ Department of Internal Medicine, Maastricht University Medical Center, Maastricht, Netherlands; ^5^ CARIM Cardiovascular Research Institute Maastricht, Maastricht University, Maastricht, Netherlands; ^6^ Centre of Expertise in Rehabilitation and Audiology, Adelante, Hoensbroek, Netherlands; ^7^ Department of Epidemiology, Johns Hopkins University, Baltimore, MD, United States; ^8^ Department Public Health and Primary Care / LUMC-Campus The Hagua, Leiden University Medical Center, Hague, Netherlands

**Keywords:** diabetes, sex disparities, risk factors, diabetes-related complications, healthcare provision, screening, systematic review

## Abstract

**Background:**

Insight in sex disparities in the detection of cardiovascular risk factors and diabetes-related complications may improve diabetes care. The aim of this systematic review is to study whether sex disparities exist in the assessment of cardiovascular risk factors and screening for diabetes-related complications.

**Methods:**

PubMed was systematically searched up to April 2020, followed by manual reference screening and citations checks (snowballing) using Google Scholar. Observational studies were included if they reported on the assessment of cardiovascular risk factors (HbA1c, lipids, blood pressure, smoking status, or BMI) and/or screening for nephropathy, retinopathy, or performance of feet examinations, in men and women with diabetes separately. Studies adjusting their analyses for at least age, or when age was considered as a covariable but left out from the final analyses for various reasons (i.e. backward selection), were included for qualitative analyses. No meta-analyses were planned because substantial heterogeneity between studies was expected. A modified Newcastle-Ottawa Quality Assessment Scale for cohort studies was used to assess risk of bias.

**Results:**

Overall, 81 studies were included. The majority of the included studies were from Europe or North America (84%).The number of individuals per study ranged from 200 to 3,135,019 and data were extracted from various data sources in a variety of settings. Screening rates varied considerably across studies. For example, screening rates for retinopathy ranged from 13% to 90%, with half the studies reporting screening rates less than 50%. Mixed findings were found regarding the presence, magnitude, and direction of sex disparities with regard to the assessment of cardiovascular risk factors and screening for diabetes-related complications, with some evidence suggesting that women, compared with men, may be more likely to receive retinopathy screening and less likely to receive foot exams.

**Conclusion:**

Overall, no consistent pattern favoring men or women was found with regard to the assessment of cardiovascular risk factors and screening for diabetes-related complications, and screening rates can be improved for both sexes.

## Introduction

In 2019, an estimated 463 million adults aged between 20 and 79 years had diabetes, affecting 9.0% of women and 9.6% of men globally. Cardiovascular diseases (CVD) are one of the most common complications of diabetes, with individuals with diabetes being two to three times more likely to develop CVD compared to those without diabetes (1). Other common diabetes-related complications include diabetic nephropathy, retinopathy, neuropathy, certain cancers, physical and cognitive impairment, depression and several types of infectious diseases (1, 2).

Although incidence rates of major CVD have been reported to be higher in men than women with and without diabetes (3, 4), there is a growing body of evidence showing that the relative risk of major cardiovascular complications conferred by diabetes is larger in women than men (2–8). Several large studies have shown that the relative risk of ischemic heart disease conferred by diabetes can be up to 50% higher in women than men (3, 5, 8). A sex differential in the consequence of diabetes has also been reported for stroke, where the relative risk of stroke was 27% higher among women than men (6). Less is known about sex differences in the effects of diabetes on microvascular complications. A meta-analysis has demonstrated that diabetes confers a 19% higher relative risk of vascular dementia in women than men (9). Sex differences have also been shown for end-stage renal disease, but not for chronic kidney disease (10).

Underlying mechanisms that explain the higher excess risk of (vascular) complications, conferred by diabetes, in women remain uncertain but may include sex disparities in the uptake and provision of healthcare (2). Currently, many guidelines on diabetes management exist. These evidence-based guidelines provide similar recommendations for both sexes on the assessment of risk factors and screening for diabetes-related complications. Therefore, throughout this systematic review, the term “disparity” will be explicitly used to refer to differences in risk factor assessment and screening for cardiovascular risk factors between men and women.

More insight in sex disparities concerning the uptake and provision of diabetes management may eventually result in more personalized diabetes care, thereby helping to further diminish the burden in both sexes. We conducted a systematic review to study whether sex disparities exist in the assessment of cardiovascular risk factors and screening for diabetes-related complications among people with diabetes.

## Methods

The protocol of this study was registered at the international prospective register of systematic reviews (PROSPERO) registry (registration number: CRD42018104414). We performed this review according to the guidelines of the preferred reporting items for systematic reviews and meta-analyses (PRISMA) (11).

### Search Strategy and Study Selection

Observational studies (including before-after studies) on the assessment of cardiovascular risk factors (HbA1c, lipids, blood pressure, BMI, and smoking status), and screening for complications (retinopathy, nephropathy, and foot ulcerations/deformities/sensory decline), in men and women with diabetes, were identified through systematically searching PubMed (1/1/2009 up to April 2020) (**Supplemental Table I**). After having identified a set of eligible studies using our search strategy, we performed manual reference and citation screening (snowballing) using Google Scholar. This method has previously been described as a good alternative to database searches once a number of eligible studies have been identified (12). Studies were included if data on the assessment of cardiovascular risk factors or screening for diabetes-related complications were provided separately for men and women. Studies presenting insufficient information about the effect size or direction of sex disparities were excluded (i.e. studies only presenting p-values). Only full-text articles written in English or Dutch were considered eligible for inclusion. Studies also including individuals without diabetes were eligible if results for individuals with diabetes were presented separately. Studies on gestational diabetes were excluded, as well as studies on which data on risk factor assessment were only adjusted for, rather than analyzed by, sex. Furthermore, studies primarily focusing on children or adolescents were excluded.

### Outcomes

The outcomes of interest were; assessment of HbA1c, lipids, blood pressure, smoking status, and BMI, screening for nephropathy, retinopathy, and performance of foot examinations, or any combination, all reported as binary variables (yes vs. no). For all outcomes of interest, we used “assessment of cardiovascular risk factors” and “screening for complications” as defined by the original article. When studies showed multiple outcome definitions, we chose the one closest to (inter)national guidelines.

### Data Collection and Management

Data extraction was performed by one author (MJ) and checked by a second author (RV). Any discrepancies between the authors during data collection were discussed with a third author (SP). The extracted data comprised: authors’ names and year of publication, country, study period, number of participants (% women), age, reported outcomes (including measures of association with corresponding confidence intervals (CIs)), and data source (**Supplemental Table II**).

### Quality Assessment

The methodological quality of the included studies was assessed by one author (MJ) and checked by a second author (RV), using a modified Newcastle-Ottawa Quality Assessment Scale for cohort studies (13). The modified scale includes six items under three categories: selection, comparability and outcome. Any discrepancies were discussed with a third author (SP).

### Data Synthesis and Analyses

It was decided beforehand not to perform any meta-analyses due to the expected heterogeneity between the included studies. Qualitative analyses were restricted to studies adjusting their analyses for age or when age was considered as an important covariable but left out from the final analyses for various reasons (i.e. backward selection). Studies only presenting crude numbers and percentages or unadjusted results are presented in **Supplemental Table III**. Where reports with overlapping study populations were found and similar outcomes of interest were studied, the study presenting data from the most recent study period or the study with most participants was included. Similarly, where studies were repeated over time, only studies with the most recent data or largest number of study participants were included. For example, the UK National Diabetes Audit is repeated every year and only data from the most recent report relevant for the outcomes of interest were extracted. Characteristics of the studies excluded from qualitative analyses are shown in **Supplemental Table IV**.

The results are presented as odds ratios (ORs) or risk ratios (RRs) with 95% CIs, with men as the reference category, unless otherwise specified. When studies only reported stratified results, e.g. by age group, ORs/RRs and the 95% CIs in each stratum were summarized using a fixed effect model. For studies that stratified the results by year, with potential overlap of included participants between strata, results from the most recent year were extracted. If studies presented multiple models, only the most extensive adjusted models were extracted. Forest plots without pooled effects were used to visualize the adjusted estimates and corresponding CIs across studies included for qualitative analysis.

## Results

**Figure 1:**
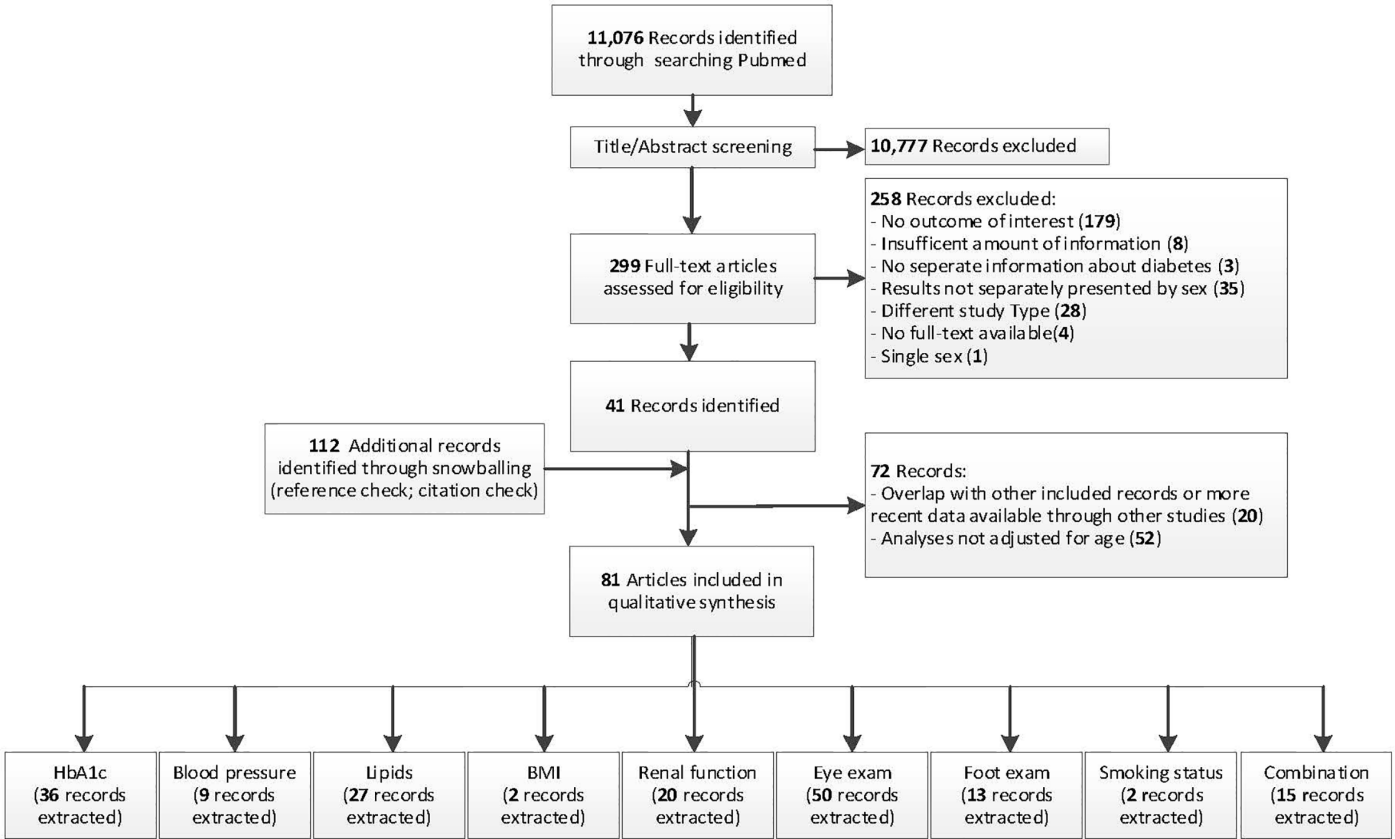
Flowchart of study selection. PubMed search was used to obtain a suitable start set for snowballing.

Overall, 81 studies were included for qualitative analyses (14–92) (**Figure 1** **)**. Characteristics of the included studies are presented in **Supplemental Table II**. The majority of studies were from Europe or Northern America (37% and 47% respectively), eight from Asia, two from Oceania, one from Africa, and one from South America. Of the 81 studies, 55 (68%) reported data on individuals with diabetes (without specifying the subtype), and 24 (30%) on individuals with type 2 diabetes. In addition, two reports from the UK National Diabetes Audit reported data on individuals stratified by diabetes subtype. Given that no other reports presented data on individuals with type 1 diabetes, only data from individuals with type 2 diabetes were extracted from the two reports. The number of included individuals per study ranged from 200 to 3,135,019. Data were extracted from various data sources (i.e. (population-based) surveys, medical records and administrative claims data) in a variety of settings, including primary care, outpatient clinics, and hospital settings.

### Risk of Bias

The risk of bias was moderate with 78% of studies showing either fair or good study quality with clearly reported information about study design, in- and exclusion criteria, data collection, and assessment of the outcome. Although most studies included a representative sample, there was considerable heterogeneity between studies with regard to the study populations making it more challenging to score this aspect **(** **Supplemental Table IV** **).**

### Assessment of HbA1c

**Figure 2:**
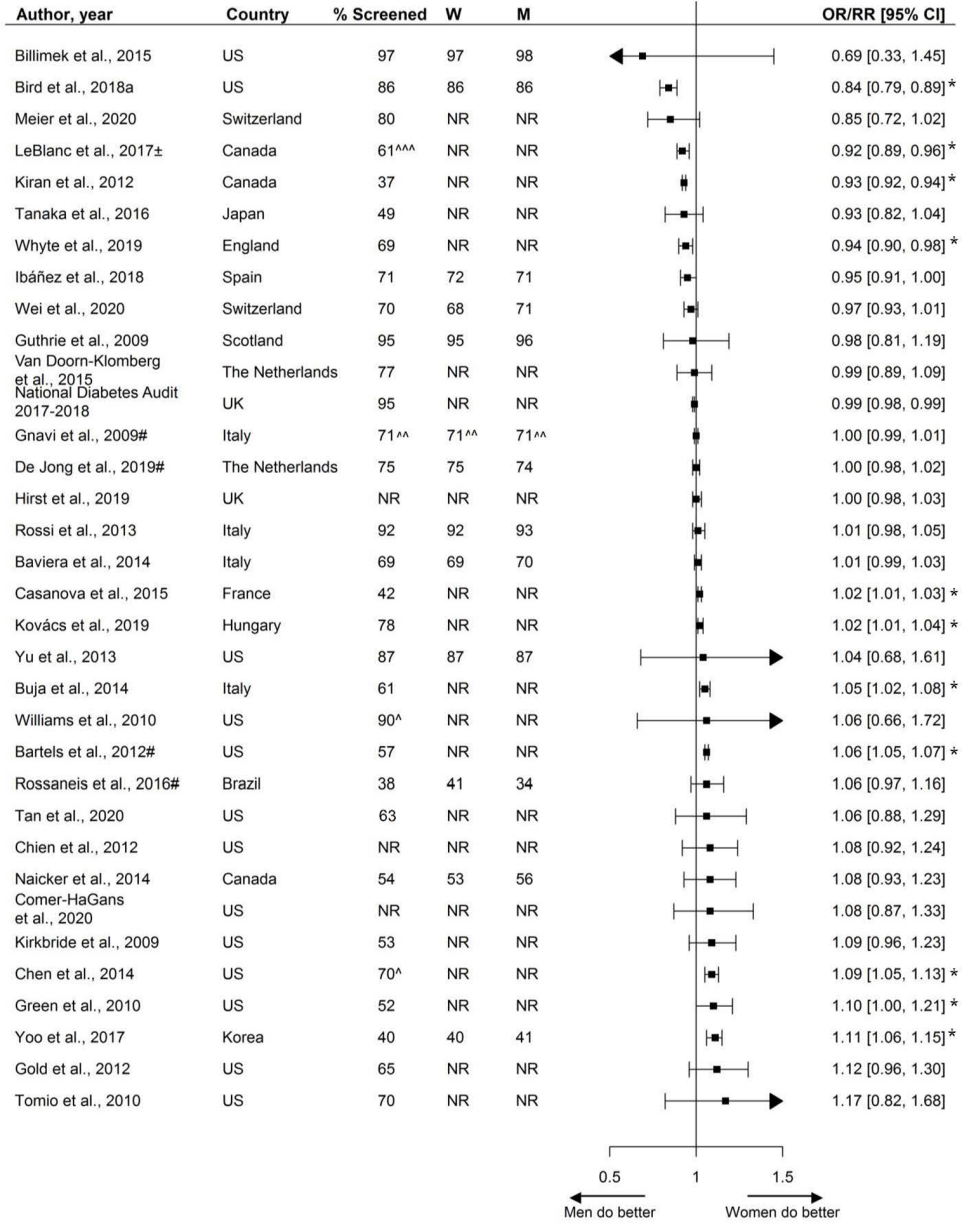
Assessment of HbA1c, expressed as adjusted odds ratios (OR) or relative risks (RR) with corresponding 95% confidence intervals (CI). Two studies are not presented in this figure because of their measure of association: Swietek et al. (33): Average Marginal Effect, (SE; p-value): −0.00031 (−0.0044; >0.05), Du et al. (92): Prevalence difference (95% CI): 3.5 (−1.0;8.0). W = % of screened women; M = % of screened men; US, United States; UK, United Kingdom; ± = 99% CI; # = Relative risk; ^ Weighted %; ^^ = Kaplan-Meyer estimates; ^^^ = Estimated %; * = statistically significant. Men = reference.

In total, 36 studies, including 6.6 million individuals, were included with median assessment rates of 74% in women and 73% in men. Most studies showed no statistically significant sex disparities in the assessment of HbA1c (70%), while 19% showed that women were more often receiving assessment of HbA1c than men, and 11% showed that men were more often receiving assessment of HbA1c than women **(** **Figure 2** **).**

### Assessment of Blood Pressure

**Figure 3:**
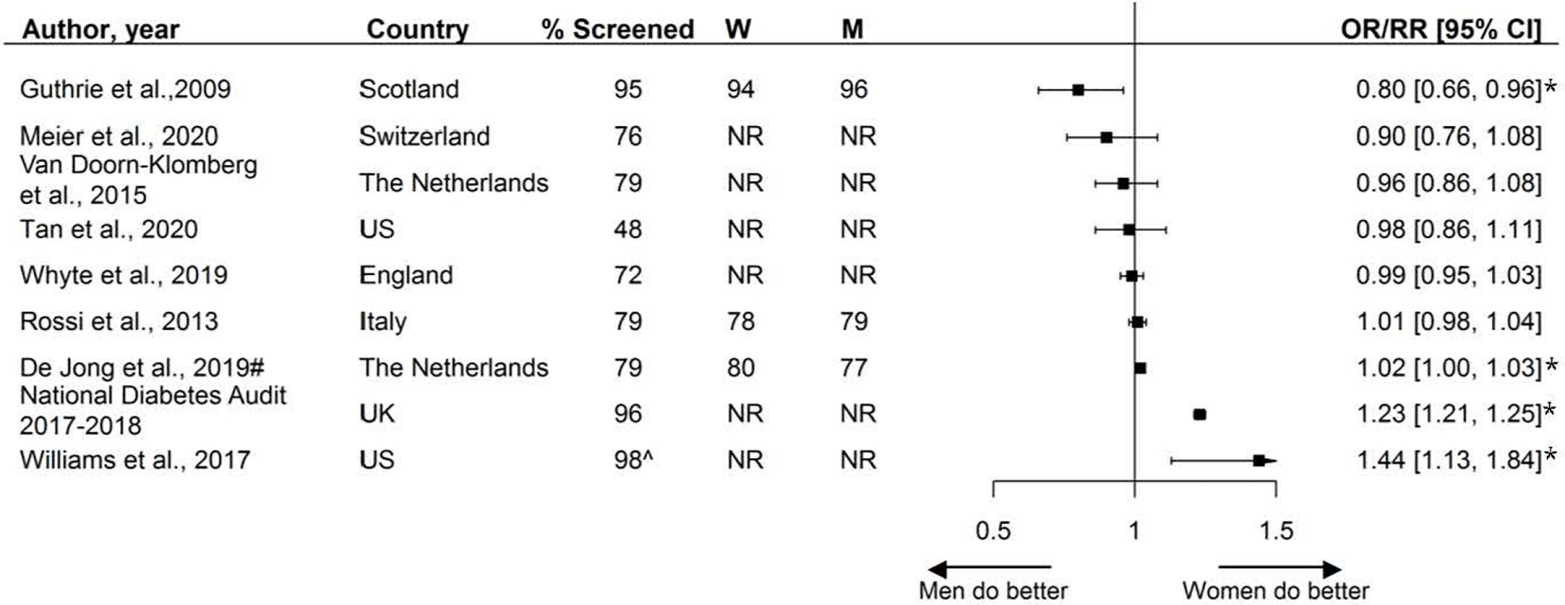
Assessment of blood pressure, expressed as adjusted odds ratios (OR) or relative risks (RR) with corresponding 95% confidence intervals (CI). W = % of screened women; M = % of screened men; US, United States; UK, United Kingdom; # = Relative risk; ^ Assumed to be weighted %; * = statistically significant. Men = reference.

The assessment of blood pressure, by sex, was reported by nine studies including 3.7 million individuals. Median assessment rate across studies was 79% (range 48% - 98%). Sex-specific percentages of blood pressure assessment were reported by three studies, ranging from 78% to 94% in women and 77% to 96% in men. Five studies showed no statistically significant disparities in the assessment of blood pressure, while three studies showed that women were more likely to receive blood pressure screening and one study reported men being more likely to receive blood pressure screening (**Figure 3** **)**.

### Assessment of Lipids

The assessment of lipids, by sex, was reported by 27 studies, including 5.4 million individuals. These studies reported on various lipid measurements, including the assessment of LDL, HDL, lipid profile, (total) cholesterol, HDL/TC-ratio, and triglycerides. Among the fifteen studies reporting the assessment of either lipids or (total) cholesterol, assessment rates ranged from 40% to 96%, with a median of 73%.

Over half the studies (8/15) reported no statistically significant or only small sex disparities, while four studies reported that, compared with men, women were less likely to receive screening, and three studies showed that women were more likely to receive screening.

Twelve studies, including data from 829,819 individuals, reported sex-specific assessment of LDL. Five studies reported that women were less likely to receive screening, four studies reported that women were more likely to receive screening than men, and the remaining three studies showed no sex disparities.

Two studies investigated sex disparities in the assessment of HDL measurements, with one reporting that women were more likely to receive screening.

**Figure 4:**
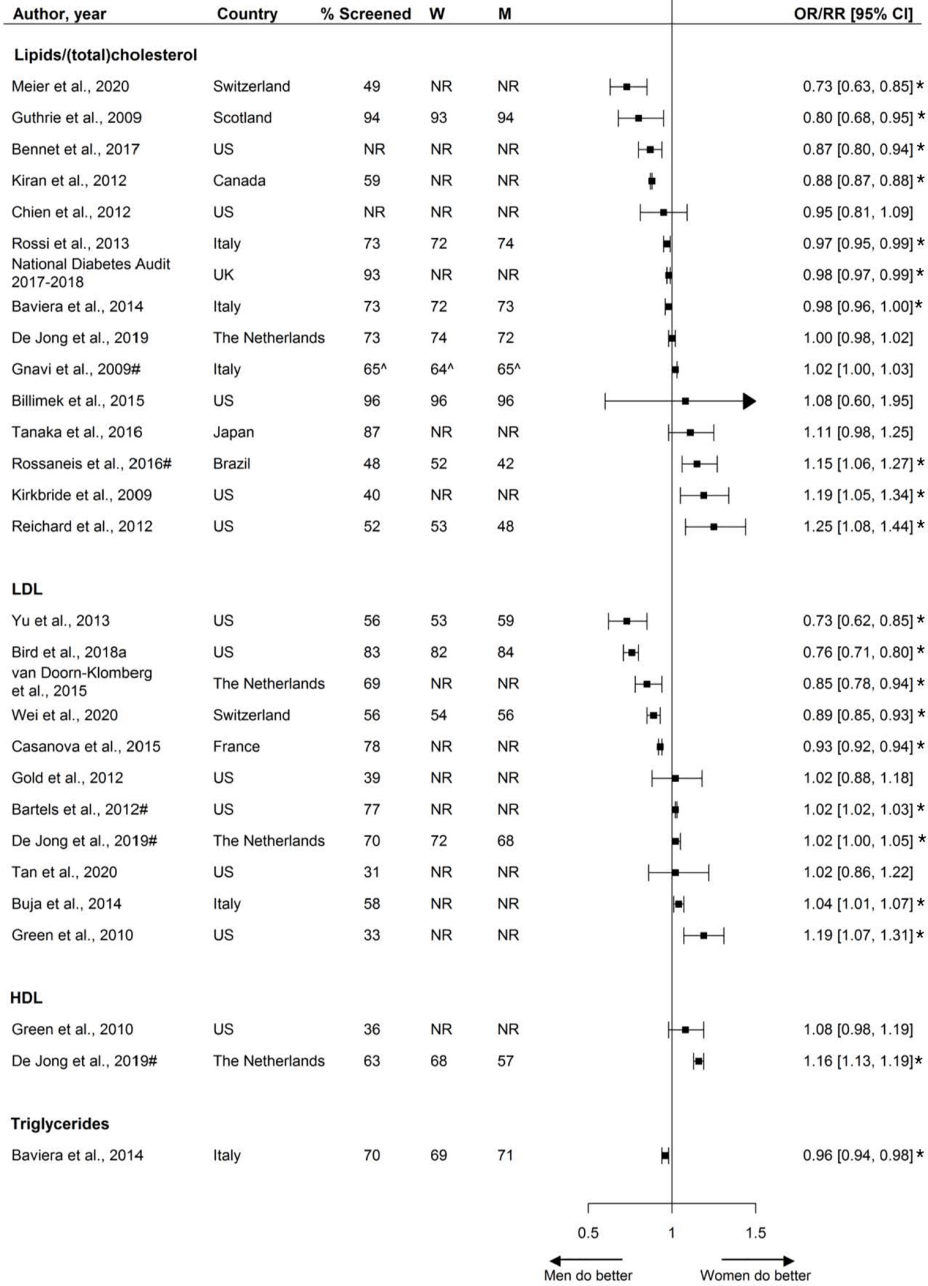
Assessment of lipids, expressed as adjusted odds ratios (OR) or relative risks (RR) with corresponding 95% confidence intervals (CI). One study is not presented in this figure because of the measure of association: Swietek et al. (33): Average Marginal Effect (LDL), (SE; p-value): 0.0045 (−0.0042; >0.05). W = % of screened women; M = % of screened men; US, United States; UK, United Kingdom; # = Relative risk; ^ = Kaplan-Meyer estimates; * = statistically significant. Men = reference.

One study reported on the assessment of triglycerides, showing that women were less likely to receive screening than their male counterparts (**Figure 4** **).**

### Assessment of BMI

**Figure 5:**
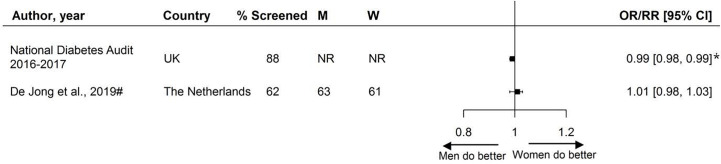
Assessment of BMI, expressed as adjusted odds ratios (OR) or relative risks (RR) with corresponding 95% confidence intervals (CI). W = % of screened women; M = % of screened men; UK, United Kingdom; # = Relative risk. Men = reference. * = statistically significant.

Two studies reported sex-specific BMI assessment; one study found that women were less likely to receive screening and the other found no sex differences (**Figure 5**).

### Nephropathy Screening

**Figure 6:**
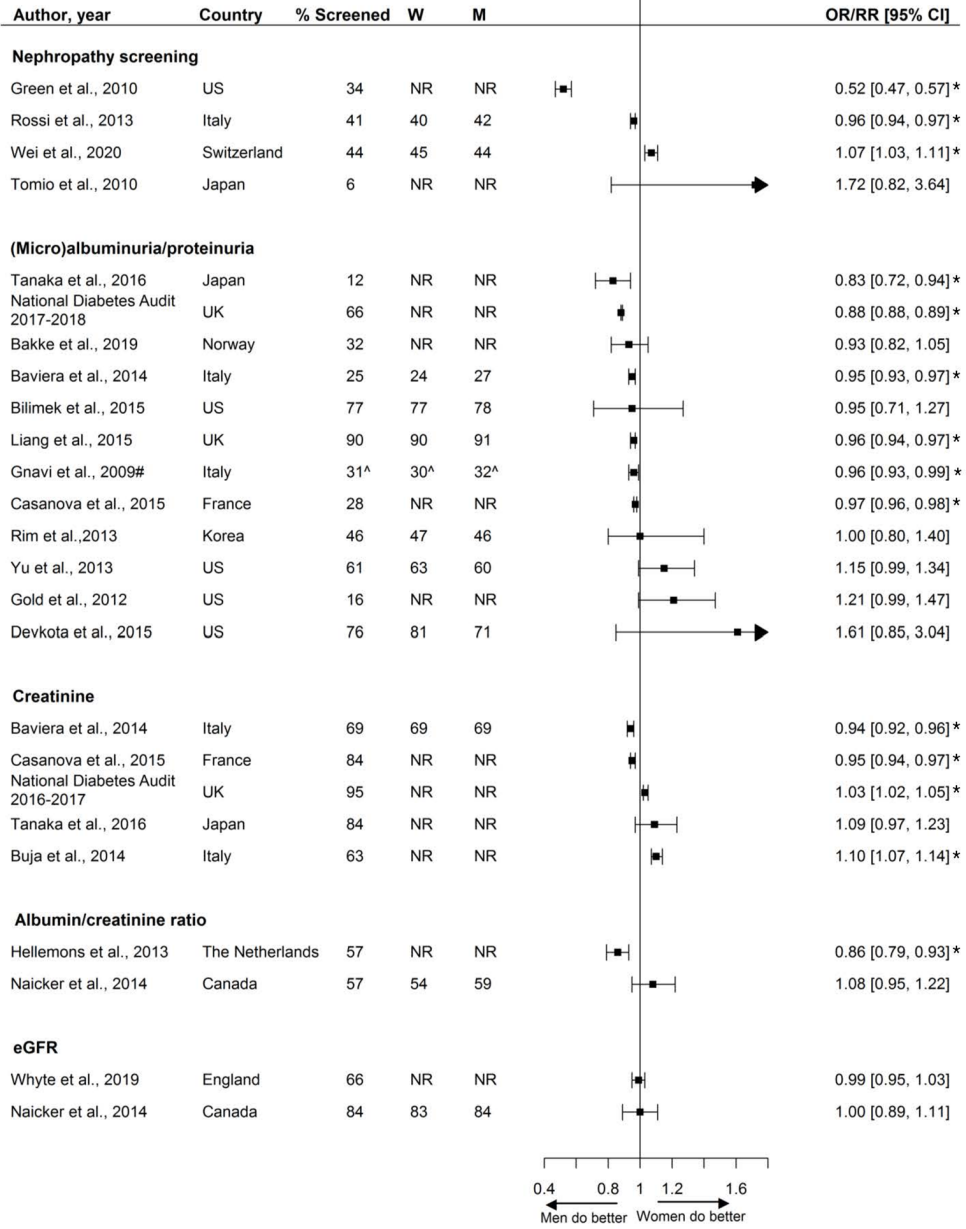
Nephropathy screening, expressed as adjusted odds ratios (OR) or relative risks (RR) with corresponding 95% confidence intervals (CI). One study is not presented in this figure because of the measure of association: Swietek et al. (33): Average Marginal Effect, (SE; p-value): −0.0073 (−0.0042; <0.05 (women less likely to receive screening). W = % of screened women; M = % of screened men; US, United States; UK, United Kingdom; # = Relative risk; ^ = Kaplan-Meyer estimate. Men = reference. * = statistically significant.

Twenty studies, including 3.9 million individuals, examined sex disparities in nephropathy screening. These studies reported on various measures to assess renal function, including estimated glomerular filtration rate (eGFR), microalbuminuria, urine albumin, albumin/creatinine ration, and serum creatinine. Two-thirds of studies reported screening rates less than 70%. Overall, there was no consistent pattern in nephropathy screening favoring either women or men (**Figure 6**).

### Retinopathy Screening

**Figure 7:**
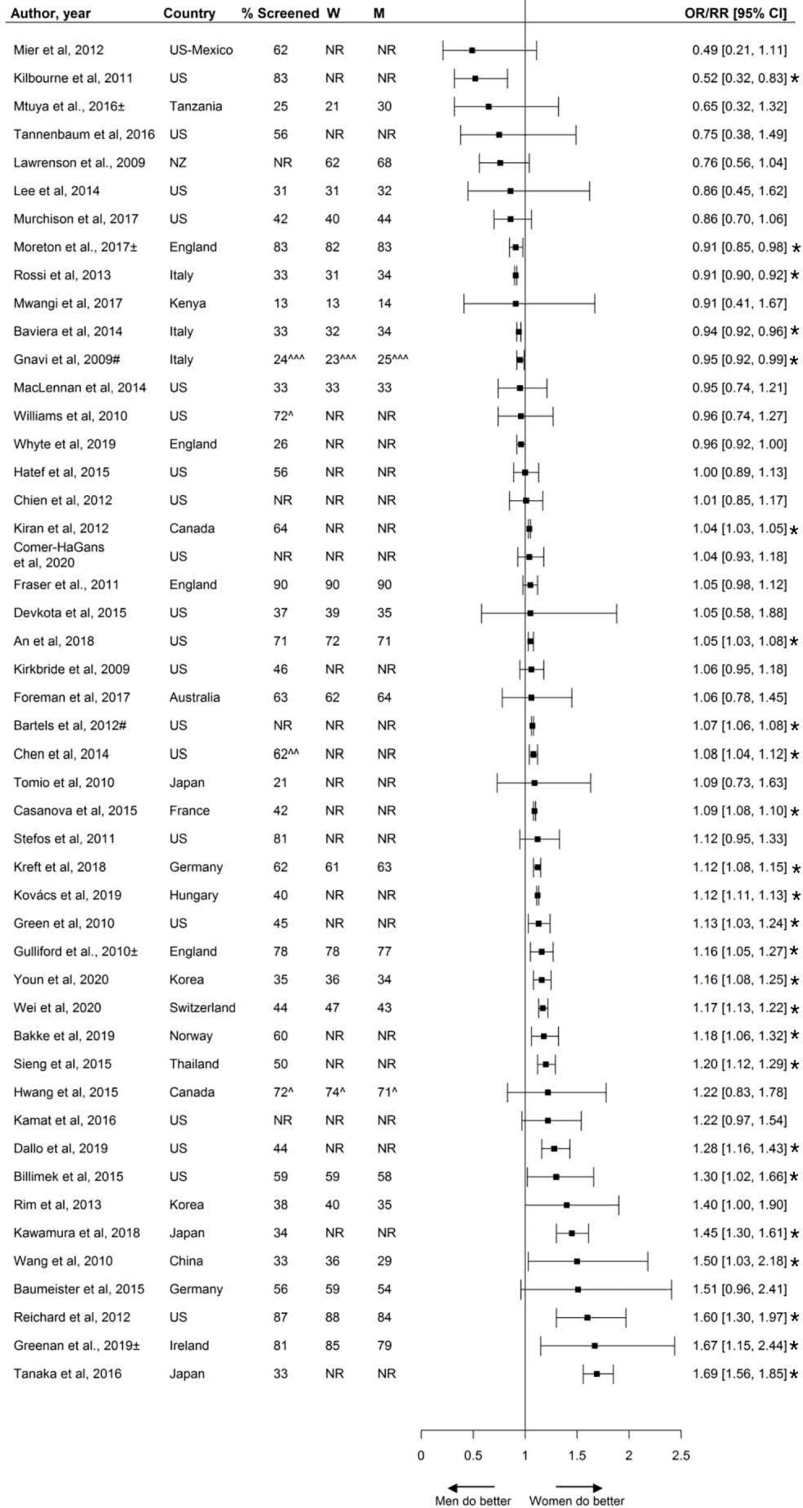
Retinopathy screening, expressed as adjusted odds ratios (OR) or relative risks (RR) with corresponding 95% confidence intervals (CI). Two studies are not presented in this figure because of their measure of association: Swietek et al. (33): Average Marginal Effect, (SE; p-value): 0.017 (−0.0043; <0.01 (women more likely to receive screening), Du et al. (92): Prevalence difference (95% CI): 12.6 (4.1;21.2). W = % of screened women; M = % of screened men; US, United States; UK, United Kingdom; # = Relative risk; ^ = 662 weighted %; ^^ = assumed to be weighted %; ^^^ = Kaplan-Meyer estimate; ± = Studies assessing screening adherence after screening invitation. Men = reference. * = statistically significant.

Fifty studies, including 3.4 million individuals, reported on retinopathy screening. Screening rates ranged from 13% to 90% across studies, with nearly half the studies reporting screening rates equal to or less than 50%. Five studies reported that women were less likely to receive retinopathy screening than men and 22 studies showed that women were more likely to receive screening (**Figure 7**).

### Foot Exams

**Figure 8:**
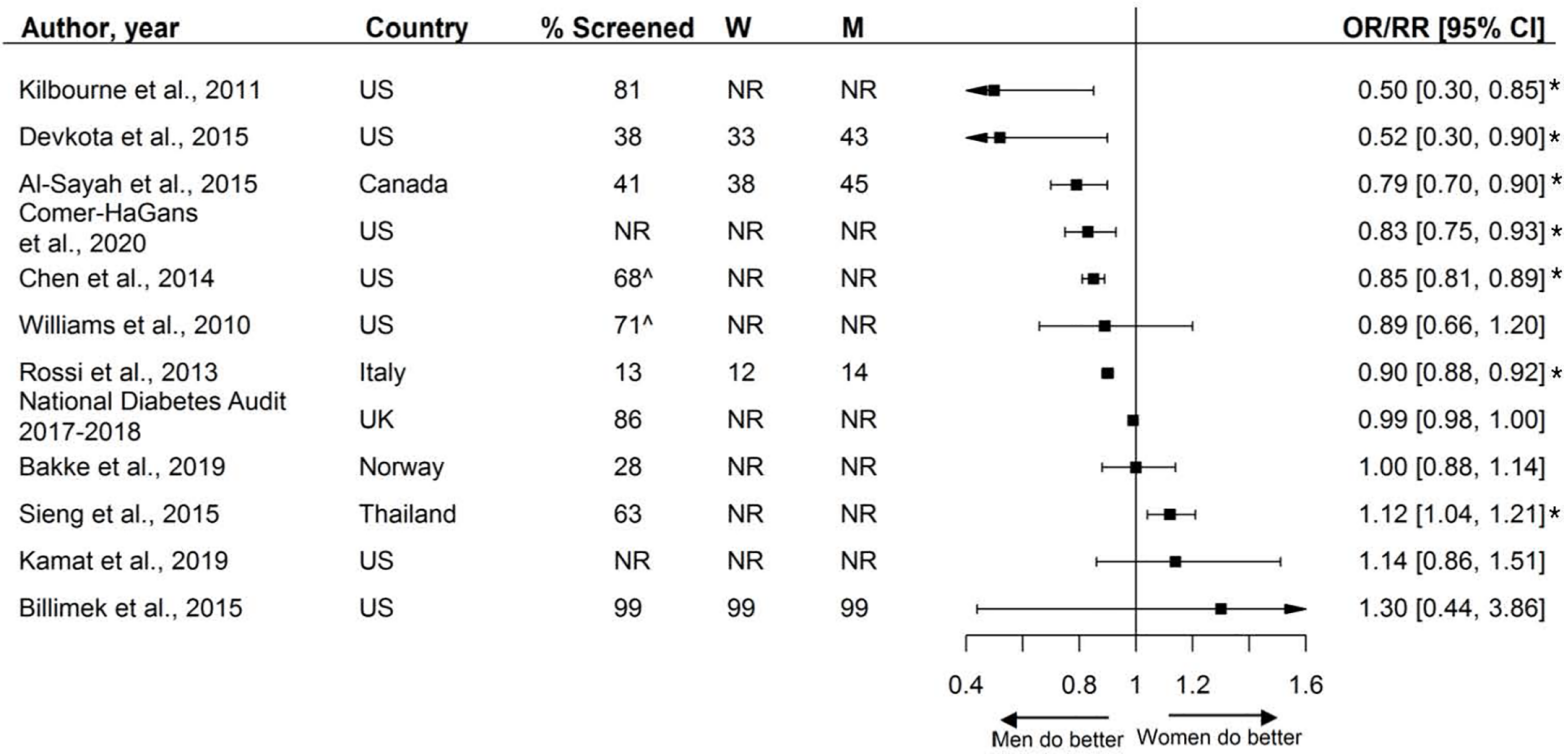
Foot exams, expressed as adjusted odds ratios (OR) with corresponding 95% confidence intervals (CI). One study is not presented in this figure because of the measure of association: Du et al., (92): Prevalence difference (95% CI 4.2 (−6.4; 14.9).W = % of screened women; M = % of screened men; US, United States; UK, United Kingdom; ^ = assumed to be weighted %. % Chen et al. extracted from the last available year. Men = reference. * = statistically significant.

Thirteen studies, including over 3.9 million individuals, reported on the sex-specific performance of foot exams. Screening rates varied from 13% to 99% across studies, with a median screening rate of 58%. Six reported that women were less likely to receive foot exams, and one study reported women being more likely to receive foot exams. The other studies reported no sex differences (**Figure 8**).

### Assessment of Smoking Status

**Figure 9:**
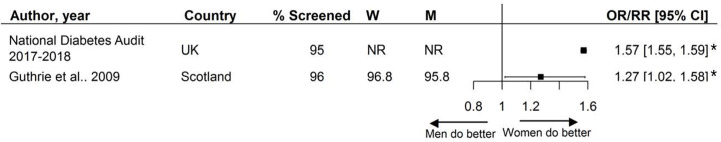
Assessment of smoking status, expressed as adjusted odds ratios (OR) with corresponding 95% confidence intervals (CI). W = % of screened women; M = % of screened men; Men = reference. * = statistically significant.

Two studies reported on the assessment of smoking status. Both studies found high screening rates (95%), and women were more likely to be screened for smoking status than men (**Figure 9**).

### Combination

**Figure 10:**
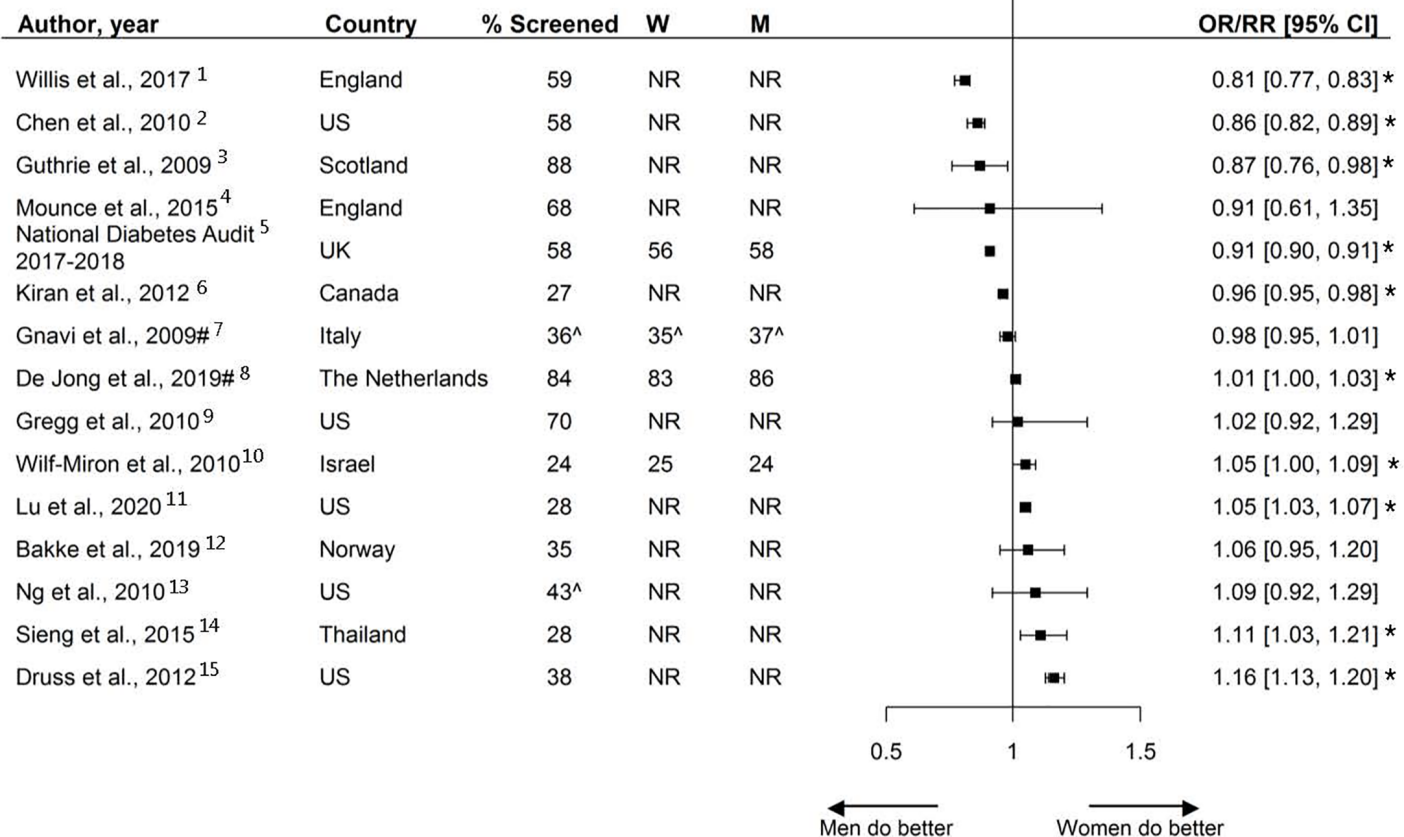
Combination of risk factor assessment and screening, expressed as adjusted odds ratios (OR) or risk ratios (RR) with corresponding 95% confidence intervals (CI). # = risk ratio; ^ = Kaplan-Meyer estimates; * = statistically significant. W = % of screened women; M = % of screened men; Men = reference. 1 = All measurements received within 12 months: blood pressure, HbA1c, cholesterol, urine albumin: creatinine ratio/protein:creatinine or proteinuria, eGFR or serum creatinine, foot and eye exams, BMI, smoking status, within 15 months (6 for HbA1c). 2 = Receiving at least 2 HbA1c measurements and 1 LDL measurement received within 12 months. 3 = All measurements received within 12 months: HbA1c, blood pressure, cholesterol, smoking status. 4 = At least one of the following measurements received within 12 months: HbA1c, proteinuria, foot exam. 5 = All measurements received within 15 months: HbA1c, blood pressure, cholesterol, serum creatinine, urine albumin, foot exam, BMI, smoking status. 6 = All measurements received within 24 months: eye exam, four HbA1c tests, and two cholesterol tests. 7 = Assessment of HbA1c and at least two measurements from among eye exams, total cholesterol, and microalbuminuria. 8 = Receiving one or more measurements within 12 months: HbA1c, blood pressure, total cholesterol, LDL, HDL, or BMI. 9 = All measurements received within 36 months: HbA1c, lipid profile, urine albumin, eye exam, and foot exam. 10 = All measurements received within 12 months: HbA1c, LDL, microalbuminuria, eye and foot exams, blood pressure and BMI. 11 = All measurements received within 12 months: HbA1c, LDL, eye exam, and medical attention for nephropathy (including screening and treatment). 12 = Receiving at least two out of three measurements: albuminuria and monofilament (foot exam) within 12 months, eye exam within 30 months. 13 = Receiving all measurements within 12 months: HbA1c, eye and foot exams. 14 = Receiving all measurements within 12 months: HbA1c, LDL, eye and foot exams. 15 = Receiving at least 2 measurements: HbA1c during 708 the measurement year, eye exam, LDL, and medical attention for nephropathy (screening test during the past year or evidence of nephropathy).

Fifteen studies reported on the assessment of a combination of risk factors and screening activities. The presence and direction of sex disparities varied across studies, with a third of the included studies reporting that, compared with men, women were less likely to receive a combination of care, one-third of studies found no sex disparities, and one-third found that women were more likely to receive a combination of care than men (**Figure 10**).

## Discussion

This systematic review including 81 studies showed that the presence, magnitude, and direction of sex disparities in the assessment of cardiovascular risk factors and screening of diabetes-related complications varied considerably across studies, with some evidence suggesting that women, compared with men, may be more likely to receive retinopathy screening and less likely to receive foot exams. In addition, only two studies reported on the assessment of smoking status; both showing that women were more likely to be screened. Overall, screening rates can be improved for both sexes.

To our knowledge, this is the first systematic review studying sex disparities in the assessment and screening of cardiovascular risk factors and diabetes-related complications among individuals with diabetes. A recent meta-analysis, including 22 studies with 4,754,782 individuals from the general population in primary care setting, showed that assessment rates of CVD risk scores and risk factors were similar between the sexes (93). In contrast to our study, the authors did find evidence of women being less likely to be assessed for smoking (93). Nevertheless, the results were comparable to our study in that no consistent pattern in risk factor assessment and complication screening favoring either men or women was found and screening rates could be improved for both sexes.

Assessment of cardiovascular risk factors and screening for diabetes-related complications is critical in guiding treatment decisions. The present study demonstrates that there is no consistent pattern in screening activities favoring men or women, suggesting that disparities in risk factor assessment and screening activities do not account for the higher relative risk of CVD conferred by diabetes previously found in women compared with men (2–8). However, other factors related to the uptake and provision of healthcare, such as treatment and adherence, may still be involved in explaining these sex differences. Although assessment of cardiovascular risk factors is one of the first steps in guiding treatment decisions, it may not necessarily be followed by equal treatment. For example, a recently published meta-analyses, including data from 2.2 million individuals in primary care, showed that women at high risk or with established CVD were less likely to be prescribed aspirin, statins, and angiotensin-converting enzyme (ACE) inhibitors, and more likely to be prescribed diuretics, than men (94). Other studies have suggested that women are less adherent to statins than men (95–97). Differences in biology may also impact women’s excess risk of CVD and it has previously been hypothesized that women experience a relatively greater increase of cardiovascular risk factor levels in the transition from normal glycaemia to diabetes (98). Differences in body anthromorphy and fat storage may be of particular interest in explaining the women’s excess risk of CVD, as fat distribution differs by sex. Sex differences in fat distribution may impact the duration of the transition from normoglycemia to overt diabetes and consequently impact the increase of other related cardiovascular risk factor levels (2).

### Strengths and Limitations

The main strength of this systematic review is the inclusion large number of studies providing sex-specific data. The majority of studies included more than 1000 individuals, of which 41 (51%) studies included over 10.000 individuals. This study also has several limitations. First, there was substantial heterogeneity between studies regarding patient population, outcome definitions, and data source and no meta-analyses were performed. Second, there was a lack of studies that specifically evaluated risk factor assessment in individuals diagnosed with type 1 diabetes. Of the studies that included individuals with diabetes without specifying the subtype, we assume that majority of the included study participants were diagnosed with type 2 diabetes. The results of this systematic review are therefore mainly applicable to those with type 2 diabetes. An appropriate method to study sex disparities separately for type 1 and type 2 diabetes would be an individual participants data (IPD) analysis, and future research should attempt to obtain individual-level patient data. Third, the majority of studies were from Europe and Northern America, thereby limiting the generalizability to other parts of the world. Fourth, screening rates varied widely between studies and across the outcomes of interest and can be improved for both sexes, nonetheless strategies on how to improve these rates are not discussed in this review. Further research is needed to explore the reasons for the suboptimal screening rates found in both sexes within the context of local and national healthcare settings.

## Conclusion

Mixed findings were found regarding the presence, magnitude, and direction of sex disparities with regard to the assessment of cardiovascular risk factors and screening for diabetes-related complications. Overall, no consistent pattern favoring men or women was found and screening rates can be improved for both sexes.

## Data Availability Statement

The original contributions presented in the study are included in the article/**Supplementary Material**. Further inquiries can be directed to the corresponding author.

## Author Contributions

RV, SP, MB, and MJ conceived the research. MJ and RV conducted the analyses and drafted the manuscript. All authors contributed critical intellectual content and made important revisions to the manuscript. RV is the guarantor of this work. All authors contributed to the article and approved the submitted version.

## Funding

This study was supported by the Netherlands Organization forHealth Research and Development (ZonMw), Gender andHealth Programme (Project no 849200001).

## Conflict of Interest

The authors declare that the research was conducted in the absence of any commercial or financial relationships that could be construed as a potential conflict of interest.
