# Supplementary material for: Sex Disparities in Cardiovascular Risk Factor Assessment and Screening for Diabetes-Related Complications in Individuals With Diabetes: A Systematic Review
Source: Front Endocrinol (Lausanne). 2021 Mar 30;12:617902. doi: 10.3389/fendo.2021.617902 (PMC8043152; doi:10.3389/fendo.2021.617902)
Supplement: Supplementary file 1 [file DataSheet_1.pdf]

Supplemental data

Supplemental table I. Search strategy and date performed.

PubMed - April 2020 *(Date restriction: 1-1-2009)*
risk factors[MeSH Terms] OR risk assessment[MeSH Terms] OR risk screening[MeSH Terms] OR health care disparity[MeSH Terms] OR cardiovascular risk management[MeSH Terms] OR risk factors[Title/Abstract] OR risk assessment[Title/Abstract] OR risk screen [Title/Abstract] OR risk screening[Title/Abstract] OR health screen[Title/Abstract] OR health screening[Title/Abstract] OR health measurement[Title/Abstract] OR health assessment[Title/Abstract] OR health care disparity[Title/Abstract] OR health care disparities [Title/Abstract] OR cardiovascular risk management [Title/Abstract] OR CVRM[Title/Abstract] OR complication screening [Title/Abstract] OR complication assessment[Title/Abstract]) OR (primary prevention[MeSH Terms] OR secondary prevention[MeSH Terms] OR primary prevention[Title/Abstract] OR secondary prevention[Title/Abstract])) OR (quality of health care[MeSH Terms] OR quality indicator, healthcare[MeSH Terms] OR guideline adherence[MeSH Terms] OR provision of health care[MeSH Terms] OR quality of health care[Title/Abstract] OR quality of care[Title/Abstract] OR quality of healthcare[Title/Abstract] OR healthcare quality [Title/Abstract] OR health care quality[Title/Abstract] OR QoC[Title/Abstract] OR quality indicator[Title/Abstract] OR quality indicators[Title/Abstract] OR quality criterion[Title/Abstract] OR quality criteria[Title/Abstract] OR guideline adherence [Title/Abstract] OR provision of healthcare[Title/Abstract] OR provision of health care[Title/Abstract] OR healthcare provision [Title/Abstract] OR health care provision [Title/Abstract])) OR (cholesterol[MeSH Terms] OR blood pressure[MeSH Terms] OR glucose[MeSH Terms] OR smoking[MeSH Terms] OR cardiovascular risk[MeSH Terms] OR diabetic complication[MeSH Terms] OR clinical care[Title/Abstract] OR cholesterol[Title/Abstract] OR low density lipoprotein[Title/Abstract] OR LDL[Title/Abstract] OR high density lipoprotein[Title/Abstract] OR HDL[Title/Abstract] OR triglycerides [Title/Abstract] OR dyslipidemia[Title/Abstract] OR hyperlipidemia[Title/Abstract] OR hyperlipidaemia[Title/Abstract] OR lipid control[Title/Abstract] OR lipid profile[Title/Abstract] OR blood pressure[Title/Abstract] OR systolic pressure[Title/Abstract] OR SBP[Title/Abstract] OR diastolic pressure[Title/Abstract] OR SBP [Title/Abstract] OR hypertension[Title/Abstract] OR bp[Title/Abstract] OR hemoglobin A1c[Title/Abstract] OR HbA1c[Title/Abstract] OR glucose [Title/Abstract] OR hyperglycemia[Title/Abstract] OR physical activity[Title/Abstract] OR smoking[Title/Abstract] OR smoker[Title/Abstract] OR body mass index[Title/Abstract] OR BMI[Title/Abstract] OR kidney function[Title/Abstract] OR diabetic kidney disease[Title/Abstract] OR nephropathy[Title/Abstract] OR renal disease[Title/Abstract] OR microalbuminuria[Title/Abstract] OR macroalbuminuria[Title/Abstract] OR albuminuria[Title/Abstract] OR glomerular filtration rate[Title/Abstract] OR GFR[Title/Abstract] OR proteinuria[Title/Abstract] OR creatinine [Title/Abstract] OR creatinine/eGFR[Title/Abstract] OR retinopathy[Title/Abstract] OR eye exam[Title/Abstract] OR eye examination[Title/Abstract] OR eye complication[Title/Abstract] OR eye complications [Title/Abstract] OR eye monitoring[Title/Abstract] OR eyes dilated[Title/Abstract] OR dilated eye exam[Title/Abstract] OR foot exam[Title/Abstract] OR foot examination[Title/Abstract] OR monofilament test[Title/Abstract] OR foot complication[Title/Abstract] OR foot complications[Title/Abstract] OR foot monitoring[Title/Abstract] OR microvascular complication[Title/Abstract] OR microvascular complications[Title/Abstract] OR macrovascular complication[Title/Abstract] OR macrovacular complications [Title/Abstract] OR vascular complication[Title/Abstract] OR vascular complications[Title/Abstract] OR cardiovascular risk [Title/Abstract] OR cardiovascular risk factors[Title/Abstract] OR CVD risk[Title/Abstract])) <br>AND   ((sex[Title/Abstract] OR gender[Title/Abstract]) AND (disparity[Title/Abstract] OR (disparities[Title/Abstract] OR difference [Title/Abstract] OR disparities[Title/Abstract] OR variation[Title/Abstract] OR variations[Title/Abstract])) OR (sex disparities[MeSH Terms]) <br>AND <br>(diabetes[MeSH Terms] OR diabetes[Title/Abstract] OR diabetic[Title/Abstract] OR DM1[Title/Abstract] OR DM2[Title/Abstract] OR DMI[Title/Abstract] OR DMII[Title/Abstract] OR T2DM[Title/Abstract] OR T1DM[Title/Abstract] OR DM) <br>NOT <br>animal

Supplemental table II. Summary of studied included for qualitative analyses.

First author, years	Country	Study period	Study size (% women) and age	Reported outcomes of interest	Primary aim & Data source
Swietek et al., 2020[1]	United States	2008-2011	82,501 (NR)<br><br>18-64	≥1 Measurements per study year: LDL, HbA1c, eye exam, nephropathy screening (medical attention for nephropathy).<br><br>*Administrative claims data*	**Primary aim:** To estimate the association between enrolment in National Committee for Quality Assurance recognized patient centered medical homes and racial disparities in quality of care for adults with major depressive disorder and comorbid medical conditions.<br><br>**Data source:** Data extracted from 2008–2011 Medicaid claims from three states with relatively high rates of Medicaid enrolment and complete claims, including those with **diabetes and major depressive disorder**. Those included were required to have >1 inpatient diagnosis or >2 outpatient or emergency department diagnoses of major depressive disorder or diabetes during a single year in the study period, and >1 claim for the condition in each year. Individuals with serious mental illnesses were excluded as well as dual enrollees in Medicare and Medicaid.
Comer-HaGans et al., 2020[2]	United States	2011-2016	13,154 (23,503,358 (51%) weighted)<br><br>20-85	≥1 Measurements per study year: Eye exam, foot exam, HbA1c.<br><br>*Self-reported*	**Primary aim:** To examine diabetes standard of care among individuals who have diabetes with and without cognitive limitation disabilities.<br><br>**Data source:** Pooled data (2011-2016) extracted from the full year Consolidated Data Files Household Component of the Medical Expenditure Panel Survey **(MEPS),** including those with **diabetes.** MEPS contains data pertaining to health care access and utilization, health care expenditures, health care satisfaction, health status, and sociodemographic data of respondents. Computer-assisted personal interviewing was used to collect the household component data.
Lu et al., 2020[3]	United States	2012	213,075 (57%)<br><br>18-64	Combination of all 4 measurements during study period (HbA1c, LDL, eye exam, nephropathy screening (including screening and treatment)).<br><br>*Administrative claims data*	**Primary aim**: To determine the extent to which the diabetes care needs are met for a population with both intellectual and developmental disabilities and diabetes who are solely insured by Medicaid in five states.<br><br>**Data source:** Administrative data from 1/1/2011 through 31/12/2012 were used to identify Medicaid members that were continuously enrolled for 11 months in 2012, with **diabetes and intellectual and developmental disabilities or diabetes only**, in 5 states (Iowa, Massachusetts, New York, Oregon and South Carolina). Individuals with dual eligibility in Medicare and Medicaid or other types of primary insurance were excluded
Wei et al., 2020[4]	Switzerland	2014	49,198 (45%)<br><br>>18 (and <75 for LDL)	≥2 HbA1 measurements, ≥1 eye exam, LDL measurements (or total cholesterol + HDL+ triglycerides), nephropathy (i.e., serum creatinine and/or albuminuria test) screening within 360 days post index date.<br><br>*Administrative claims data*	**Primary aim:** To describe regional variation in the utilization of the four measures across small regions in Switzerland and to explore potential influencing factors.<br><br>**Data source:** Data extracted from health insurance claims provided one of the largest health insurance companies in Switzerland. Those enrolled with Helsena with **diabetes** who were prescribed any diabetes medication between 1/1/ 2014 and 27/12/ 2014 were included. Date of the first prescription of any diabetes medication in 2014 (incident diabetes) or January 1, 2014 (prevalent diabetes) was considered as the index date for each participant. Those with incomplete insurance coverage in 2014 or not surviving until the end of 2014 were excluded, as well as those living outside Switzerland, asylum seekers, Helsana employees, with incomplete address information, living in nursing homes with lump-sum reimbursement.
Youn et al., 2020[5]	Korea	2015 (survey year)	20,904 (48%)<br><br>≥19	≥1 Eye exams within the year prior to the survey.<br><br>*Self-reported*	**Primary aim:** To investigate the uptake rate variance of fundus examination for diabetes-related complications among demographically and geographically diverse communities and examine determinants that influence this rate focusing on outpatient eye care clinic accessibility at community level.<br><br>**Data source:** Data on individual-level factors was extracted from the nationwide 2015 Community Health Survey including information about the uptake of retinal screening within the prior year among those with **diabetes**.
Tan et al., 2020[6]	United States	1/1/2015 – 31/12/2018	4,552 (53%)<br><br>≥18	≥1 HbA1c, blood pressure, or LDL measurements between 6 months prior and post index date.<br><br>*Electronic medical records*	**Primary aim:** To examine the potential sociodemographic disparities in type 2 diabetes management and care among adult individuals, after controlling for clinical and behavioral factors.<br><br>**Data source:** Data extracted from a linked database of the National Health and Wellness Survey and a large ambulatory electronic health record database (EHR). The index date was the date when individuals completed. Those that completed the survey between 2015-2018; with ≥1 clinical measurements; a diagnosis of **type 2 diabetes** in the survey or EHR or ≥1 oral glucose-lowering prescription in the EHR; and ≥12-month

					follow-up in the EHR database were included. Individuals with type 1 or gestational diabetes were excluded.
Meier et al., 2020[7]	Switzerland	2018 (baseline date)	3,833 (43%)<br><br>NS	≥1 Measurements within 12 months prior to baseline date: HbA1c, blood pressure, cholesterol.<br><br>*Electronic medical records*	**Primary study aim:** To describe quality indicator performance in diabetes care in Swiss primary care and to analyze associations of practice, general practitioner and patient covariates with quality indicator performance.<br><br>**Data source:** Baseline data extracted from an electronic medical record database collected within a cluster randomized controlled trial. The baseline assessment covered 12 months retrospectively using electronic medical records database of the Institute of Primary Care of the University of Zurich. Those diagnosed with **diabetes** ≤4 months before the baseline date were eligible for inclusion.
Hirst et al., 2019[8]	United Kingdom	1/1/2005-31/12/2014	100.000 (45%)<br><br>NS	No HbA1c measurements within 12 months post previous measurement.<br><br>*Electronic medical records*	**Primary aim:** To examine whether both an individual's previous HbA1c and the reporting deadline at the end of the administrative year are associated with over-frequent or delayed HbA1c testing in national data in the UK, and whether there are regional disparities across the UK and whether other pre-defined participant or general practitioner practice level variables may be associated with very frequent or delayed HbA1c testing intervals.<br><br>**Data source:** Data extracted from those with **diabete**s randomly selected from the Clinical Practice Research Datalink (CPRD) over a 10-year period (1/1/2005-31/12/2014). CPRD is a governmental database providing anonymized data from UK primary care. For those with existing diabetes, baseline HbA_{1c} test was defined as first HbA_{1c} test after 1/1/2005. Included participants had ≥2 HbA_{1c} tests prior to the baseline test and post diagnosis. People with incident diabetes during follow-up, and ≥3 HbA_{1c} test post diagnosis, were included in the analysis. For those, the baseline test was the second test. Participants had ≥2 HbA_{1c} tests for inclusion. Those with gestational diabetes, malnutrition related diabetes, maturity-onset diabetes of the young, <3 HbA1c measures in total, steroid-induced diabetes or haemochromatosis-related diabetes, cancer or end-stage renal disease, were excluded.
Bakke et al., 2019[9]	Norway	(1/7/2012-31/12/2014)	8,246 (45%)<br><br>≥18	≥1 Measurements within 15 months prior to 31/12/2014: albuminuria, foot exam; ≥1 eye exams within 30 months prior to 31/12/2014, combination (≥2 out of 3)<br><br>*Electronic medical records*	**Primary aim:** To assess population, general practitioner, and practice characteristics associated with the performance of microvascular screening procedures and to propose strategies to improve type 2 diabetes care.<br><br>**Data source:** Data extracted from electronic health records from general practices located in five of Norway's nineteen counties with urban and rural areas participating in the ROSA 4 study, including adults with **type 2 diabetes** who had their main follow-up in general practice and a diabetes duration of ≥1 year. Those diagnosed with diabetes in 2014, new to the general practitioner, with main follow-up at by a specialist, in nursing homes, with unknown list-holding general practitioner, type 1 diabetes including LADA, and other i.e. MODY, pancreatitis, or undetermined were excluded.
Dallo et al., 2019[10]	United States	2015	6,622 (54%)<br><br>≥18	No eye exam during study period.<br><br>*Administrative data*	**Primary aim:** To estimate and compare the management of diabetes among Arab, Asian, non-Hispanic Black, and non-Hispanic Whites attending a large health system in metropolitan Detroit.<br><br>**Data source:** Data extracted from a primary care sample of patients with **diabetes** within a health system in metropolitan Detroit.
De Jong *et al.,* 2019[11]	The Netherland s	2013	12,512 (50%)<br><br>≥20 to <100	≥1 Measurements during study period: HbA1c, blood pressure, total cholesterol, LDL, HDL, BMI, combination (≥1).<br><br>*Electronic medical records*	**Primary aim:** To evaluate whether there are sex disparities in cardiovascular risk management in patients with diabetes in primary care.<br><br>**Data source:** Data extracted from a longitudinal primary care medical record database (Julius General Practitioners Network) of general practices in Utrecht and vicinity (The Netherlands), including those with a diagnosis of **diabetes** before the study period with continuous enrolment during study period.
Whyte et al., 2019[12]	England	1/1/2012-31/12/2016	49,380 (44%)<br><br>≥18	Uninterrupted annual monitoring during study period: HbA1c, blood pressure, eGFR, eye exam.<br><br>*Electronic medical records*	**Primary aim:** To evaluate contemporary data as to whether disparities exist in glycaemic control, monitoring, and prescribing in people with type 2 diabetes.<br><br>**Data source:** Data extracted from the Royal College of General Practitioners Research and Surveillance Center database. Those diagnosed with **type 2 diabetes** prior to 2012 and continuance in the database over the study period were eligible for inclusion.
Du *et al.,* 2019[13]	Germany	2008-2011	526 (43%)<br><br>40-79	≥1 Measurements within prior 12 months: HbA1c, eye exam, foot exam.<br><br>*Self-reported*	**Primary aim:** To study gender disparities in cardiovascular risk profiles and diabetes care based on a nationwide representative sample of adults with type 2 diabetes in Germany.

					**Data source:** Data extracted from the German National Health Interview and Examination Survey (DEGS1 2008-2011), including a nationwide representative sample of adults with type 2 diabetes. **Type 2 diabetes** was defined as a history of physician-diagnosed diabetes or current use of antidiabetic medication, excluding those with type 1 and gestational diabetes.
Kovács et al., 2019[14]	Hungary	2015	478,660 (NR)<br><br>≥18	≥1 Measurements during study period: HbA1c, Eye exam.<br><br>*Administrative data*	**Primary aim:** To evaluate the influence of general medical practice characteristics on performance indicators.<br><br>**Data source:** Data extracted in December 2015 from general practices that provide primary healthcare to adults. Data for the analyses were provided by the National Institute of Health Insurance Fund Management (NIHIFM). NIHIFM established a nationally integrated system of health care indicators with financial incentives in 2010. Individuals with d**iabetes** receiving glucose-lowering medication were eligible for inclusion.
Greenan et al., 2019[15]	Ireland	11/2013-5/2015 (data extraction)	1,200 (33%)<br><br>≥12	Eye screening attendance after referral/invitation (attending all screening and treatment appointments)<br><br>*Medical records*	**Primary study aim:** To determine whether geodemographic factors, specifically age, gender or commuting distance, affect the attendance rates of patients referred to a Diabetic Retinopathy Treatment Centre from the Irish National Diabetic Retinal Screening Programme.<br><br>**Data source:** Data extracted from the first 1200 patients with **diabetes** who were referred for ophthalmic assessment between 11/2013 and 5/2015 to Cork University Hospital's diabetic retinopathy treatment clinic from the diabetic retinopathy screening program (Diabetic RetinaScreen). In Ireland, the National Diabetic Retinal Screening was introduced in 2013. It offers annual screening and treatment where necessary to all patients with diabetes aged 12 years and older currently living in Ireland.
Kamat et al., 2019[16]	United States	1999-2016 (survey period)	7,521 (NR) (49% assumed to be weighted)<br><br>>20	≥1 Measurements prior 12 months to survey: foot exam, eye exam.<br><br>*Self-reported*	**Primary aim:** To examine trends and disparities in the quality of diabetes care among US adults with diabetes.<br><br>**Data source:** Data extracted from the National Health and Nutrition Examination Survey (NHANES) 1999-2016, including those with self-reported **diabete**s (not during pregnancy) based on questions about physician diagnosed diabetes and medication use, and levels of fasting glucose or HbA1c. Survey respondents were selected using a complex, stratified, multistage probability sampling design of the US noninstitutionalized civilian population. Survey data were gathered through in-home interviews, physical exams, and lab tests.
An et al., 2018[17]	Unites States	1/1/2009-31/12/2010 (inclusion period and index date) 31/12/2013 (follow-up)	204,073 (48%)<br><br>≥18	≥1 exams each 12 month period from the index date if retinopathy is present and ≥1 exams each 24 months if no retinopathy is present.<br><br>*Electronic medical records*	**Primary aim:** To assess long-term adherence, in patients with diabetes, to the recommended regular eye exam guidelines, and to determine factors associated with non-adherence.<br><br>**Data source:** Patient data extracted from Kaiser Permanente Southern California (KPSC). KPSC is a non-profit, integrated health-care delivery organization in Southern California. KPSC provides integrated, comprehensive medical services within its own facilities, which include hospitals, outpatient facilities, and a centralized laboratory. All aspects of care and interaction with the health-care delivery system are captured in a continuously updated electronic Organization. Those with ≥2 outpatient-visits with a diagnosis code for **diabetes** between 1/1and 2009 and 31/12/2010 were included and the first diagnosis of diabetes or dispense date of an antidiabetic drug was defined as the index date. Those without continuous health-plan membership or drug benefit during the 12 months before and after the index date were excluded, as well as those with gestational diabetes.
Ibáñez et al., 2018[18]	Spain	15/5/2014 (data extraction)	32,206 (44%)<br><br>≥20	≥1 Measurements 15 months prior to data extraction: HbA1c.<br><br>*Electronic medical records*	**Primary aim:** To determine if achievement of control targets in patients with type 2 diabetes was associated with personal socioeconomic factors and if these associations were sex-dependent.<br><br>**Data source:** Data extracted from individuals with a diagnosis of **type 2 diabetes** on 15/5/2014 registered in Atena. Atena is a Primary Care Electronic Medical Record System containing information from all individuals with type 2 diabetes managed by the Regional Health Service of Navarre (northern Spain).
Bird *et al.*, 2018a[19]	United States	2013-2014 (1 year)	78,529 (49%)<br><br>NS	NO Measurements during study period: HbA1c, LDL.<br><br>*Administrative claims data*	**Primary aim:** To quantify persistent gender gaps in cardiovascular risk management and to assess the performance of routinely used commercial population health management tools in helping systems narrow gender gaps.<br><br>**Data source:** Anonymized data of medical and pharmacy claims, laboratory results, and enrolment data from one national health plan for commercial health plan members

					drawn from a population across Atlanta, Houston, New York City/Northern New Jersey and Southern California. Those with **diabetes** were included.
Kreft et al., 2018[20]	Germany	2004-2014	26,560 (51.6%)<br><br>≥50	≥1 Eye exams during study period.<br><br>*Administrative claims data*	**Primary aim:** To assess factors associated with diabetic retinopathy screening uptake following a diagnosis of type 2 diabetes in Germany.<br><br>**Data source:** Data extracted from randomly sampled members of the largest German public health insurance. Data from persons born prior to 1955 who first experienced diagnosis of **type 2 diabetes** during the study period and living in private households and institutions was obtained. Medical individual-level data for all members was registered and collected quarterly from the beginning of 2004 until end of 2014, or earlier study exit. Those with chronic eye disease which necessitated regular ophthalmic check-ups, age-related macular degeneration or other macular disease, or retinopathy present in the quarter before the first type 2 diabetes diagnosis were excluded.
Kawamura et al., 2018[21]	Japan	1/2005 - 3/2013	6,492 (34%)<br><br>≥20	≥1 eye exams within one year of initial drug therapy (from the index month).<br><br>*Administrative claims data*	**Primary aim:** To investigate the influence of comorbidities on undergoing a diabetic eye exam in patients with newly diagnosed type 2 diabetes.<br><br>**Data source:** Data extracted from health insurance claims made between 1/2005 and 3/2013 using the database of Japan medical Centre Ltd. (Tokyo, Japan). This database consists of beneficiaries in health insurance unions across Japan in 2012, including individuals diagnosed with **type 2 diabetes** between 1/2005 and 3/2013 that had been prescribed antiglycaemic drugs with a 12-month follow-up from the index month. The index month was defined as the first month in which the study patients had been diagnosed with type 2 diabetes and received antiglycaemic drugs. Those who were not prescribed antidiabetic drugs after the index month were excluded, as well as those diagnosed with diabetes or prescribed antidiabetic drugs during the nine months after registration in the database, with diabetic retinopathy<br>prior to the index month, those who had undergone eye exams, who had been diagnosed with eye diseases, or who had undergone an intervention for the eyes within the six months preceding the index month, in order to select patients who did not visit the ophthalmologist regularly. Lastly, those without information regarding the facility at which diabetes treatment took place in the index month were excluded.
National Diabetes Audit[22]<br>*(3 separate reports)*	England and Wales	2017-2018, 2016-2017; 2012-2013	Varies per audit period with up to 3,135,019 (44%) individuals in 2017-2018<br><br>≥12 (HbA1c: All)	Varies per subtype and audit period, including ≥1 measurements during study period (15 months): HbA1c, blood pressure, cholesterol (triglycerides and another type of fat in the blood), creatinine, urine albumin, foot, smoking, BMI, combination (all eight care processes (excl. eye exam (<12 years only HbA1c)).<br><br>*Administrative data*	**Primary aim:** To measure the effectiveness of diabetes healthcare against NICE Clinical Guidelines and NICE Quality Standards, in England and Wales.<br><br>**Data source:** Administrative data extracted from participating general practitioners via pre-agreed extracts of their computer system and specialist diabetes service units in secondary care hospitals. This includes data from children being treated in adult care settings; but does not cover pediatric units. Both previously diagnosed and newly diagnosed individuals with **type 2 diabetes** during the audit period were included. General practices were invited to participate in the audit through their clinical systems. The audit operates under an 'opt in' model to remain open and transparent with practices and services about what data are being collected. Data from 2012-2013 (measurement of creatinine) included individuals with **'all' diabetes**.
Foreman et al., 2017[23]	Australia	3/2015-4/2016 (recruitment)	1,076 (55%)<br><br>≥40 (indigenous)<br>≥50 (non-indigenous)	≥1 Eye exam (indigenous within prior 12 months, non-indigenous within prior 24 months).<br><br>*Self-reported*	**Primary aim:** To determine adherence to National Health and Medical Research Council (NHMRC) eye examination guidelines for Indigenous and non-Indigenous Australian people with diabetes."<br><br>**Data source:** Indigenous and non-Indigenous Australians with self-reported **diabetes** were recruited and examined between 3/2015 and 4/2016 after a multistage, random cluster sampling approach selecting 30 geographic sites in the five mainland Australian states and the Northern Territory; recruiters went door to door to recruit the included Indigenous and non-Indigenous Australians .During the interview participants were asked whether they had seen an ophthalmologist or optometrist for a diabetic eye examination, and if so, how long ago (in years). This information was used to determine the proportion of participants who adhered to the NHMRC guidelines.
Mwangi et al., 2017[24]	Kenya	NR	270 (53%)<br><br>≥18	≥1 Eye exams in prior 12 months.<br><br>*Self-reported*	**Primary aim:** To identify the demand-side factors that influence uptake of eye examination among patients already utilizing diabetes services in three counties of Kenya.<br><br>**Data source:** Data extracted from patient surveys. A three-stage sampling, strategy was used to select counties, diabetes clinics, and patients with **diabetes** attending these

					clinics. Patients were selected by random sampling from the people attending the clinic on the day of interview. Those with diabetes, residents in the county, and receiving care at the participating clinics were eligible for inclusion. Acutely ill individuals were excluded.
LeBlanc et al., 2017[25]	Canada	1/42005-31/3/2009 and 1/4/2010-31/3/2014	83,580 (52%)<br><br>≥20	≥2 HbA1c measurements per year.<br><br>*Administrative data*	**Primary aim:** To evaluate the influence of the introduction of a pay-for-performance program implemented in 2010 for family physicians on the glycaemic control of patients with diabetes.<br><br>**Data source:** Data extracted from 5 administrative databases from the New Brunswick Department of Health before (2005-2009) and after (2010-2014) the implementation of a pay-for-performance program implemented for family physicians on the glycaemic control of those with **diabetes**. Included were those in the province with diabetes if the detection of their diabetes occurred between April 1995and March 2014 and eligible participants had to be followed by family physicians paid by fee-for-service. Data was extracted by matching Medicare patient list with glycaemic control data from Laboratory Data Repository. Additional information was extracted from Medicare Resident Registry and the Physician Profile database.
Yoo et al., 2017[26]	Korea	1/1/2013-31/12/2013	43,283 (47%)<br><br>NS	≥2 HbA1c measurement during study period.<br><br>*Administrative claims data*	**Primary aim:** To analyze compliance to HbA1c testing guidelines and explore associated individual and area-level determinants, focusing on regional variation.<br><br>**Data source:** Data extracted from the Korean National Health Insurance **(**KNHI) Research Database. The KNHI is a mandatory universal health insurance in Korea. Individuals included had claims for **diabetes** in 2013 and were prescribed any antidiabetic medications, including insulin, in 2012. Those who were hospitalized during 2013 were excluded, as well as those who had made only one claim for diabetes over the year and those who died in 2013.
Bennet et al., 2017[27]	United States	2007-2012	Unclear<br>NS	≥1 Cholesterol measurements prior 12 months per survey.<br><br>*Self-reported*	**Primary aim:** To examine service utilization among persons with selected disabling conditions and diabetes, compared to those without.<br><br>**Data source:** Data extracted from 2007-2012 Medical Expenditure Panel Survey Full-Year Consolidated files (MEPS), medical conditions files, and the 1996-2012 pooled linkage files. MEPS sample is derived from the National Health Interview Survey, which is the primary survey that collects information regarding the health of the US civilian, non-institutionalized population. MEPS respondents are followed for two years, and overlap with subsequent panels on 6-month intervals, including those with **diabetes.**
Williams et al., 2017[28]	United States	2002-2011	17,702 (56.4%) (17,857,174 weighted)<br><br>≥18	≥1 Measurements prior 12 months per survey: blood pressure<br><br>*Self-reported*	**Primary aim:** To assess disparities in quality of care indicators in a nationally representative sample of men and women with diabetes.<br><br>**Data source:** Data extracted from the Medical Expenditure Panel Survey Household Component (MEPS-HC) from 2002-2011, including individuals with self-reported **diabetes.** MEPS is a survey of a nationally representative US civilian, non-institutionalized population and is administered by the Agency for Healthcare Research and Quality. Data from 10 years were pooled for this study. The MEPS sample is drawn from reporting units in the previous year's National Health Interview Survey, a nationally representative sample with oversampling for non-Hispanic Blacks and Hispanics of the U.S. civilian, non-institutionalized population.
Willis *et al.*, 2017[29]	England	1/12012-31/3/2013	25,816 (46%)<br><br>≥13	Combination (all: blood pressure, HbA1c, cholesterol, urine albumin:creatinine ratio (or protein:creatinine testing, or proteinuria), eGFR (or creatinine), foot exam, eye exam, smoking, BMI) measured during study period (6 months for HbA1c).<br><br>*Electronic medical records*	**Primary aim:** To examine the extent to which variations in achievement to high impact indicators can be explained using routinely collected data.<br><br>**Data source:** Routinely collected, anonymized electronic primary care data from a sample of general practices in West Yorkshire (England). Data covered the period 1/1/2012 to 31/3/2013, and were extracted during April 2014. Those with **type 2 diabetes** receiving care at one of the participating practices that are using a specific computerized patient record are included.
Murchison et al., 2017[30]	United States	1/1/2007-12/12/2010	1,967 (55%)<br><br>>40	Follow-up eye exam <15 months for mild or no diabetic retinopathy, <12 months for moderate diabetic retinopathy and <4 months from the index visit	**Primary aim:** To evaluate individual factors that impact adherence to eye care follow-up in patients with diabetes.<br><br>**Data source:** Data extracted using billing and administrative information, including those who had their initial visit to a general ophthalmology or retina clinic within an urban academic eye hospital between 1/1/2007 and 31/12/2010. Patient charts were

				for severe diabetic retinopathy.<br><br>*Medical records*	reviewed to determine additional clinical information and confirm eligibility. The index visit was defined as the date of the first dilated fundus exam in this eye care system, including a diagnosis of **type 1 or type 2 diabetes** or diabetic retinopathy. Patients who did not have a documented dilated fundus exam at the designated eye clinics within 30 days of type 1/type 2 diabetes or diabetic retinopathy noted in their billing records were excluded. The diagnosis of diabetes did not have to be new to the patients.
Moreton *et al.,* 2017[31]	England	1/4/2012 – 30/4/2013	21,753 (43%)<br><br>≥12	Eye screening attendance after invitation.<br><br>*Screening program records*	**Primary aim:** To investigate variables at the demographic and primary care practice levels that influence the uptake of diabetic retinopathy screening.<br><br>**Data source:** Data extracted from the Oxfordshire Diabetic Eye Screening Programme management software, including those with **diabetes** newly referred to the screening program and those invited in previous years. The analysis was restricted to the first date of invitation for each registered person from 1/4/2012 until 30/4/2013
Tanaka et al., 2016[32]	Japan	4/2011-3/2012	11,500 (NR)<br><br>20-69	≥1 Measurements during study period: Eye exam, microalbuminuria, creatinine, any lipid test (total cholesterol, LDL, HDL or triglycerides), HbA1c (≥1 per 3 months).<br><br>*Administrative claims data*	**Primary aim:** To investigate the process quality of diabetes care provided to patients under universal health insurance coverage.<br><br>**Data source:** Data extracted from health insurance claims data, managed by the Japan Medical Data Center Claims Database. Beneficiaries with **type 2 diabetes** covered by Health Insurance Societies between 4/2010 and 3/2012 that regularly visited clinics or hospitals at least every 3 months in the identification year (4/2010-3/2011) were included. Those with insulin-dependent diabetes were excluded, as well as those that dropped out of care during follow-up. Only those who made follow-up visits were included and patient adherence to follow-up visits during study period was assessed.
Rossaneis et al., 2016[33]	Brazil	NR	1,515 (63%)<br><br>≥40	HbA1c NOT measured in prior 6 months, lipid profile (triglycerides, total cholesterol, HDL, and LDL) NOT measured in prior 12 months.<br><br>*Assumed to be self-reported*	**Primary aim:** To investigate disparities with regard to foot self-care and lifestyle between men and women with diabetes.<br><br>**Data source:** Data extracted from a sample of individuals with **type 2 diabetes** living in the urban area of a large city in the South of Brazil. Study participants were drawn among those enrolled in the Hypertensive and Diabetics Individuals Registration System. Individuals selected were invited to participate in the study and data were collected at primary health care services through patient interviews and medical chart extraction. Those undergoing dialysis, with active ulcers in the lower limbs, without cognitive capacity, or not willing to participate were excluded.
Tannenbaum et al., 2016[34]	United States	20/2011-9/2013 (Survey period)	264 (57%)<br><br>≥40	≥1 Eye exam 12 months prior to the survey.<br><br>*Self-reported*	**Primary aim:** To examine the prevalence and correlates of eye screening adherence among select Hispanics/Latinos living with diabetes.<br><br>**Data source:** Data extracted from an ancillary study of the Hispanic Community Health Study/Study of Latinos (HCHS/SOL) (Miami site). HCHS/SOL is an ongoing multisite study of prevalence of and risk factors for disease among Hispanics/Latinos. Participants included Hispanics/Latinos who underwent a baseline examination/risk factor assessment (4/3/2008-30/6/2011) and then completed a survey on vision health/knowledge (10/ 2011-9/ 2013). Diabetes status was clinically determined at the baseline study. Those with **diabetes** were included.
Mtuya et al., 2016[35]	Tanzania	4/2013 – 6/2013 (interview period)	203 (57%)<br><br>NS	Follow-up eye exam after referral in the period between initial exam and interview.<br><br>*Self-reported*	**Primary aim:** To assess the proportion of patients not presenting for follow-up and the reasons for poor follow-up of diabetic patients after screening for retinopathy in Kilimanjaro Region of Tanzania.<br><br>**Data source:** The study was carried out under the auspices of the Kilimanjaro Diabetic Programme (KDP). KDP screens **diabetic patients** for retinopathy at diabetic clinics throughout the Kilimanjaro Region. KDP visits each diabetic clinic regularly where enrolled patients are screened with a mobile retinal camera. Following screening, patients are either sent a text message or are phoned 2–4 weeks after their screening event and informed that further investigations and possibly treatments are needed. Patients are advised whether they should attend within 1 month or within 3 months depending on the severity of their retinopathy. Patients who have normal results or do not need further investigations are advised to attend another screening event after 1 year. The study was carried out between 4/2013 and 6/2013. Patients were considered eligible if they had their screening event in 2012 and if they had been referred to KCMC eye department after their screening event. Patients were categorized as non-attenders at follow-up if they had not attended KCMC Hospital when the interviews were conducted. In 2012, 1106 patients were screened by the KDP for diabetic retinopathy. Of these, 420 had retinopathy requiring further assessment and were recommended to attend a follow-up appointment at KCMC. The researchers randomly selected 294 of these patients for interview through a simple random sampling technique. The selected patients were contacted using details stored on the KDP

					database and were interviewed at their local hospital during subsequent KDP screening events.
Hatef et al., 2015[36]	United States	2010 and 2012	8,902 (69%)<br><br>18-64	Annual eye exam.<br><br>*Administrative claims data*	**Primary aim:** To assess how well a managed care organization performed annual diabetic eye screening in a Medicaid population, and to identify barriers to completion.<br><br>**Data source:** Healthcare claims data for Medicaid patients with **diabetes** covered by Priority Partners Managed Care Organization with continuous enrolment during measurement year in 2010 and 2012 were collected. Annual rates for eye exams in those years were reported. In 2011 the Johns Hopkins HealthCare instituted its program to increase the completion rate for annual diabetic eye exams.
Baumeister et al., 2015[37]	Germany	2008-2012 (survey periods)	456 (44%)<br><br>20-81	Eye exam within 12 months prior to the survey.<br><br>*Self-reported*	**Primary aim:** To study trends of barriers to receiving recommended eye care among subjects with diabetes aged 20-81 years in northeast Germany.<br><br>**Data source:** Data extracted from the Study of Health in Pomerania (SHIP-Trend), consisting of a population-based samples of adults from West Pomerania, a north-eastern German region. SHIP-Trend was conducted between 2008 and 2012. Those with self-reported **diabetes** were included.
Sieng et al., 2015[38]	Thailand	1/4/2012-30/6/2012 (collection of records)	26,869 (70%)<br><br>≥35	≥1 Measurements in prior 12 months: LDL, foot exam, eye exam, HbA1c (≥2), combination (all).<br><br>*Medical records*	**Primary aim:** to compare the process of diabetes care of specialist diabetes clinics, and general medical clinics for different hospital level (regional, provincial, and community).<br><br>**Data source:** Data for this study were obtained from an ongoing project "An assessment on quality of care among patients diagnosed with type 2 diabetes and hypertension visiting hospitals of Ministry of Public Health and Bangkok Metropolitan Administration in Thailand, 2011-2012". A proportional to size stratified cluster sampling approach was used to collect medical record data of patients **with type 2 diabetes**, diagnosed for at least 12 months, from all provinces in Thailand. Data were collected retrospectively by reviewing medical records for patients attending clinics from April 1 to June 30, 2012.
Mounce et al., 2015[39]	England	2010-2011 (survey period)	907 (47%)<br><br>≥50	Combination (not receiving ≥1 assessments: HbA1c, proteinuria (in those without established renal disease and no ACE inhibitor or angiotensin II receptor blocker) and foot exam) within 12 months prior to the survey.<br><br>*Self-reported*	**Primary aim**: To determine which patient characteristics were associated with failure to receive indicated care for diabetes over time.<br><br>**Data source:** Data extracted from the English Longitudinal Study of Ageing (ELSA), including adults with **diabetes**. ELSA is a longitudinal cohort study of adults living in private households in England. Beginning in 2002–3, participants were followed up with two-yearly 'waves' of data collection. The original cohort was drawn from households that had previously responded to the Health Survey for England (HSE) in either 1998, 1999 or 2001. Replenishment cohorts were added in 2006–7 (sampled from HSE 2001–2004) and 2008–9 (sampled from HSE 2006) to correct for the original sample ageing and loss to follow-up. The cohort is intended to be representative of older people living independently in England. Data collection took place via face-to-face interviews in participants' homes, with additional information collected during a nurse visit in 2008–9. For this study survey responses about quality indicators from the 2010-2011 wave was explored. Descriptive characteristics used for modelling achievement of care in 2010-2011 were obtained from the 2008-2009 wave.
Liang et al., 2015[40]	United Kingdom	2007-2012 (cohort entry)	65,790 (42%)<br><br>>50	Proteinuria (Urine albumin, microalbumin or protein test) at any time during follow-up.<br><br>*Electronic medical records*	**Primary aim:** To describe proteinuria monitoring in patients with type 2 diabetes.<br><br>**Data extraction:** Data extracted from UK Clinical Practice Research Datalink, including patients with **type 2 diabetes** with first antidiabetic drug use in 2007–2012. Cohort entry was defined as the date of the first ever antidiabetic drug prescription. ≥1 year of registration before and after cohort entry was required. Those with a diagnosis of type 1 diabetes, gestational diabetes mellitus, diabetes insipidus, or secondary or other forms of diabetes, including nutritional, genetic, postsurgical, and drug-induced or chemical-induced diabetes, at any time during study period were excluded.
Hwang et al., 2015[41]	Canada	2011 (survey year)	2,323 (NR) (1,324,553 (42%) Weighted)<br><br>≥20	≥1 Eye exams within prior 2 years from survey.<br><br>*Self-reported*	**Primary aim:** To examine the association between socioeconomic factors and ophthalmic care services/visual impairment among patients with diabetes.<br><br>**Data source:** Data extracted from the Survey on Living with Chronic Disease in Canada (SLCDC)–Diabetes Component 2011. SLCDC is a survey focusing on the experiences of Canadians living with chronic health conditions. Non-institutionalized individuals with self-reported physician **diagnosed type 2 diabetes** on the 2010 Canadian Community Health Survey were invited to participate in the 2011 SLCDC-DM survey. Full-time members of the Canadian Forces and residents of First Nations Reserves, Crown lands, institutions, and the 3 territories were excluded.

Casanova et al., 2015[42]	France	2008 and 2011	142 291 (47%) and 166 896 (47%)<br><br>≥18	≥1 Annual measurements: Eye exam, LDL, creatinine, microalbuminuria, HbA1c (≥3).<br><br>*Administrative claims data*	**Primary aim:** To assess the evolution of paraclinical monitoring of patients with type 2 diabetes between 2008 and 2011.<br><br>**Data source:** Data extracted from the Provence-Alpes-Côte-d'Azur (PACA) regional health insurance reimbursement database (national health insurance fund), including individuals with **type 2 diabetes** living in PACA and who had 3 or more reimbursements for diabetes medications during the 12 months before the start of each study period.
Devkota et al., 2015[43]	United States	9/2008-8/20011 (chart review)	350 (54%)<br><br>≥22	Annual eye exam, microalbuminuria (or ACEI or ARB prescription), foot exam<br><br>*Electronic medical records*	**Primary aim:** To determine whether meeting diabetes quality indicators improves as general internal medicine physicians' progress from first to last year of residency.<br><br>**Data source:** Chart review from electronic health records of **type 2 diabetes** patients who visited internal medicine residency clinics from 9/2008 to 8/2011. Charts were selected by resident provider year (year 1, 2, and 3).
Billimek *et al.,* 2015[44]	Unites States	5/2006 - 6/2011	1,369 (59%)<br><br>≥18 to <80	≥1 Measurements 12 months prior to baseline: HbA1c, lipid profile, microalbuminuria, foot exam, eye exam.<br><br>*Medical records*	**Primary aim:** To examine whether disparities in quality of care, intensity of lipid-lowering medication regimen and medication adherence explain gender disparities in dyslipidemia.<br><br>**Data source:** Data extracted from the observational component of the R2D2C2 study, enrolling a sample of individuals from 7 outpatient clinics affiliated with an academic medical centre. The patient sample was drawn from a diabetes registry representing adults with a diagnosis of **type 2 diabetes** who had ≥1 encounters with family medicine, internal medicine or endocrinology within a 12 month period. All participants completed a baseline questionnaire. Medical records were abstracted for the 12-month period leading up to the date the questionnaire was completed.
Al-Sayah et al., 2015[45]	Canada	12/2011-12/2013 (recruitment period)	2,027 (45%)<br><br>≥18	≥1 Exams during the past year: feet checked for sores or irritations<br><br>*Self-reported*	**Primary study outcome:** To examine the prevalence and predictors of foot disease, self-care and clinical monitoring in adults with type 2 diabetes in Alberta, Canada.<br><br>**Data source:** Baseline data extracted from the Alberta's Caring for Diabetes complications study, including adults with **type 2 diabetes**. Individuals were recruited over a 2-year period (12/2011-12/2013) through primary care networks, diabetes clinics and various forms of public advertisements. Those with gestational diabetes or type 1 diabetes were excluded.
Van Doorn-Klomberg et al., 2015[46]	The Netherland s	2010 (data extraction)	11,178 (50%)<br><br>≥18	≥1 Measurements in 12 months: HbA1c, systolic blood pressure, LDL.<br><br>*Electronic medical records*	**Primary aim:** To assess the strength of associations between patient factors and diabetes care processes and outcomes.<br><br>**Data source:** Routinely collected data of those with **diabetes** in 59 participating Dutch primary care practices was extracted. All participating practices extracted the data in 2010. The extraction included information from all contacts with a time window of one year.
Lee et al., 2014[47]	United States	2007-2008 (inclusion period) through 2010	200 (54%)<br><br>NS	≥1 Eye exam every 15 months<br><br>*Electronic medical records*	**Primary aim:** To estimate the prevalence of, and factors associated with, eye exam guideline compliance among patients with diabetes, but without diabetic retinopathy.<br><br>**Data source:** Data extracted from computerized billing records database, including those with **diabetes** receiving care at the Bascom Palmer Eye Institute and residing within the same county as the screening facility. The sample of available and eligible patient records first seen in 2007–2008 was reviewed for demographic information at the screening visit, and all clinic visits through 2010 were ascertained by chart review. Those not receiving eye screening every 15 months were contacted to check whether they received care at different locations. For those of who it remained unclear whether they received screening every 15 months were classified as not receiving screening. Those with diabetic complications, retinopathy or any other eye disease were excluded.
MacLennan et al., 2014[48]	United States	2007 (inclusion period)	867 (62%)<br><br>>18	≥1 Eye exam within 1 year post index date.<br><br>*Electronic medical records of the billing and accounting system*	**Primary aim:** To investigate eye care utilization among patients with diabetes who are seen in a county hospital clinic in the South that primarily serves high risk low income patients who are predominantly non-Hispanic African Americans.<br><br>**Data source:** Data extracted from two years of follow-up data, to examine eye care utilization among **diabetes** patients seen in 2007 at the internal medicine clinic of a large, urban, county hospital that serves primarily low income, non-Hispanic African American patients(Birmingham Alabama). The date of their first clinic visit in 2007 was defined as an index date. Follow-up (retrospectively) was carried out by linking patients' personal identifiers, i.e., medical record numbers, to electronic records of the hospital's billing and accounting system which included dates and procedures of patient encounters in the hospital's ophthalmology clinic. Those with ophthalmic complications were excluded.

Buja *et al.*, 2014[49]	Italy	2009	105,987 (48%)<br><br>≥16	≥1 Measurements during study period: HbA1c, creatinine, LDL.<br><br>*Administrative data*	**Primary aim:** To ascertain the prevalence of diabetes in an Italian population, stratified by age, gender and citizenship; and to identify rate of compliance with recommended guidelines for monitoring diabetes, to see whether disparities exist in the quality of diabetes management.<br><br>**Data source:** Anonymized data extracted from the VALORE project. The dataset was obtained by processing public health administration databases and included those registered with general practices in six Italian regions including individuals with **diabetes** diagnosed before 1/1/2009. Those lost to follow-up during 2009 were excluded.
Naicker *et al.,* 2014[50]	Canada	2008, 2009, 2010	2,343 (53%)<br><br>≥40	Test NOT recommended over a course of 12 months: HbA1c (<2), eGFR (<1), albumin/creatinine-ratio (<1).<br><br>*Electronic medical records*	**Primary aim:** To determine whether any sex disparities exist in adherence to process of care guidelines for cardiovascular disease within primary care practices in Ontario, Canada.<br><br>**Data source:** Data extracted from pooled cross-sectional baseline data collected through a larger improvement initiative known as the Improved Delivery of Cardiovascular Care study. Individuals at high CVD risk (i.e. **diabetes**) or prevalent CVD and receiving primary care across eastern Ontario (Canada) at participating practices were included. Data on guideline adherence was obtained through baseline chart abstraction and represent patient-level guideline adherence rates prior to intervention.
Baviera *et al.,* 2014[51]	Italy	20002– 2006	158,426 (45%)<br><br>40 - 89	≥1 Measurements per year: HbA1c, cholesterol (total, LDL, HDL), triglycerides, creatinine, eye exam, microalbuminuria.<br><br>*Administrative data*	**Primary aim:** To investigate whether sex-related disparities exist in terms of management and hospitalization in patients with newly diagnosed diabetes.<br><br>**Data source:** Data extracted using linkable administrative health databases of the Lombardy (Italy) region, including the regional database, pharmacy prescription database, and hospital discharge database. Individuals with **diabetes** were included if they had not been diagnosed with diabetes within the previous 2 years. Laboratory tests and special medical exams were recorded from 2002 to 2006. All participants were followed until the first hospitalization for cardiovascular reason, death, emigration, admission to a nursing home, or until 31/12/2007.
Chen et al., 2014[52]	United States	2001-2010 (Survey period)	355,620 (41%) (50% weighted)<br><br>≥18	≥1 Measurements 12 months prior to survey: Eye exam, foot exam, HbA1c (≥2).	**Primary aim:** To examine trends in the receipt of eight recommended diabetes clinical and self-care indicators from 2001 to 2010 and assess racial/ethnic disparities in care.<br><br>**Data source:** Data extracted from the 2001 to 2010 Behavioral Risk Factor Surveillance System (BRFSS). BRFSS is a telephone survey in which self-reported, health-related data are collected monthly in all 50 states, the District of Columbia, Puerto Rico, the US Virgin Islands, and Guam. Those with self-reported physician diagnosed **diabetes** were included, and gestational diabetes was excluded.
Rim et al., 2013[53]	Korea	2005, 2007-2009 (survey period)	1,671 ()<br><br>≥40	≥1 Measurements 12 months prior to survey: eye exam, microalbuminuria.<br><br>*Self-reported*	Data extracted from the third (2005) and fourth (2007-2009) Korean National Health and Nutrition Examination Survey (KNHANES). KNHANES is a nationally representative survey to estimate the health and nutritional status of the Korean population and consisted of a health interview, health examination survey, and nutrition survey. Those with diabetes were included in this study. Those with **diabetes** diagnosed before the age of 40 were excluded as well as those with missing data for certain socio-demographic factors.
Yu *et al.*, 2013[54]	United States	2001 – 2002	4,839 (49%)<br><br>NS	≥1 Measurements up to 12 months prior to baseline survey:<br>HbA1c, LDL, microalbuminuria (≥1 up to 24 months prior to baseline survey)<br><br>*Assumed to be self-reported*	**Primary aim:** To examine the associations between sex and selected diabetes process of care measures and self-care activities in a cohort of primary care patients with diabetes.<br><br>**Data source:** Data extracted from a population of primary care patients from the PATHWAY study, including those with **diabete**s at Group Health, a non-profit health maintenance organization in Washington and Idaho (US). It maintains a registry of the individuals with diabetes and their guideline recommended test results. Nine primary care clinics were chosen for patient recruitment based on the number of diabetic patients, ethnic diversity, and proximity to Seattle. Those with gestational diabetes, cognitive impairment, severe illness, deceased, disenrolled, or with language or hearing problems were excluded.
Rossi *et al.*, 2013[55]	Italy	2009	415,294 (45%)<br><br>NS	≥1 Measurements during study period: HbA1c, lipid profile (LDL or total cholesterol and HDL and triglycerides), blood pressure, nephropathy exam, foot exam, eye exam.	**Primary aim:** To investigate the quality of type 2 diabetes care according to sex.<br><br>**Data source:** Anonymized data using the Italian Association of Clinical Diabetologists Annals. Clinical data collected during the year 2009 were extracted from electronic medical records. Only those with **type 2 diabetes** were included.

				Electronic medical records	
Hellemons et al., 2013[56]	The Netherlands	2007-2009	14,120 (52%)<br><br>NS	Albumin/creatinine ratio measurements each calendar year.<br><br>*Electronic medical records*	**Primary aim:** To evaluate guideline adherence and factors associated with albuminuria screening and treatment in type 2 diabetes patients in primary care.<br><br>**Data source:** Data extracted from electronic medical records from primary practices using the Groningen Initiative to Analyze Type 2 diabetes Treatment database. The patient population for this study consisted of all those who had been diagnosed with **type 2 diabetes** for at least 1 year on 1/1/2007, with continuous enrolment until 7/2010. Guideline adherence was evaluated in the years 2007-2009.
Mier et al., 2012[57]	US–Mexico border area	2008 (survey period)	249 (66%)<br><br>≥65	≥1 Eye exams within 12 months prior to the survey.<br><br>*Self-reported*	**Primary study aim:** To determine the level of health care access for older Hispanics with type 2 diabetes living in a US–Mexico border area, and personal and health correlates to health care utilization.<br><br>**Data source:** Data obtained by community-based assessment conducted in 2008 at a clinic, senior centers, and colonias. Colonias are impoverished neighborhoods with substandard living conditions along the US–Mexico border. The health assessment included Hispanics with **type 2 diabetes**, living in Hidalgo County, Texas, at the Texas–Mexico border. To maximize recruitment, certified community health workers recruited participants in both clinical and community settings, including: a federally-qualified community health clinic that provided services for the uninsured and low-income individuals, and 2 nonclinical-based settings (community senior centers and colonias).
Druss et al., 2012[58]	United States	2003-2004	118,190 (64%)<br><br>≤65	Combination (≥2 measures: HbA1c during measurement year, eye exam, LDL, or nephropathy screening (either screening during past year or evidence of nephropathy).<br><br>*Claims data*	**Primary aim:** To study the impact of mental comorbidity on quality of diabetes in a national sample of Medicaid enrollees.<br><br>**Data source:** Data extracted from fee-for-service Medicaid enrollees with Diabetes during 2003-4. (across 50 states) Eligible where those with continuous enrolment for at least 1 year, ≥2 encounters for diabetes in an outpatient setting, or ≥1 inpatient encounter with **diabetes** related ICD-9 Codes, and ≥1 claim with any **mental disorder** excluding organic conditions such as Dementia and Delirium. Those with dual eligibility were excluded, as well as those with managed care claims.
Bartels et al., 2012[59]	United States	2006	256,331 (61%)<br><br>≥65	≥1 Measurements during study period: LDL, eye exam, HbA1c (≥2)<br><br>*Claims data*	**Primary aim:** To examine how the presence of Rheumatoid arthritis affected HbA1c and lipid measurement in older adults with diabetes.<br><br>**Data source:** Data extracted from a random national sample of 2004 to 2005 Medicare patients. Eligible were those with **diabetes** who were continuously enrolled and alive from 2004 to 2006. Beneficiaries without continuous Medicare Part A or B coverage, or those enrolled in a Medicare health maintenance organization or railroad benefits were excluded, as well as those encounters during 2004 to 2006.
Chien et al., 2012[60]	United States	2003-2007	5,557 (66%)<br><br>≥18	Annual HbA1c, lipids, eye exam.<br><br>*Administrative claims data*	**Primary study aim:** To evaluate the impact of a "piece-rate" pay for performance (P4P) program aimed at improving diabetes care processes, outcomes and related healthcare utilization for patients enrolled in a not-for-profit Medicaid-focused managed care plan.<br><br>**Data source:** Data extracted from the Hudson Health Plan, which is a not-for-profit Medicaid-focused managed care health plan serving the Hudson Valley region of New York. Late in 2003 Hudson piloted a **diabetes** improvement initiative in 6 of 118 participating practices. This program targeted members who were missing one or more of the following clinical tests: HbA1c, LDL cholesterol, dilated retinal exam, and microalbuminuria. At that time, providers were offered $100 for each patient completing all the missing care processes. A revised program was launched 8/ 2004. In the beginning of 2005, the program was revised a second time such that incentive amounts in 2005 P4P incentive were 3 times that offered in 2003 and more than twice the 2004 bonus. Each March, Hudson generated patient reports identifying adult enrollees with diabetes and any care elements that were missing or below national goals. Hudson representatives hand-delivered final reports and payments to physician practices and were available to discuss results and identify opportunities for improvement; additional follow-up and coaching occurred at 2, 4, and 6 weeks later. Analyses were restricted to those who were continuously enrolled in Hudson for ≥ 6 months.
Kiran et al., 2012[61]	Canada	1/4/2006-31/3/2008	734,974 (NR)<br><br>≥40	≥1 Eye exams, ≥4 HbA1c measurements, ≥2 cholesterol tests and combination (all) over 2-year study period.	**Primary aim:** To assess the impact of a diabetes incentive code introduced for primary care physicians in Ontario, Canada, in 2002 on quality of diabetes care at the population and patient level.

				Administrative claims data	**Data source:** Administrative data was extracted from Ontarians with **diabetes** (diagnosed ≤31/8/2006) to examine the use of the code and receipt of three evidence-based monitoring tests from 2006 to 2008. The researchers assessed testing rates over time, and before and after billing of the incentive code. Patients were excluded if they were not assigned to a primary care physician, when residing in long-term care facilities, or when registered with the OHIP after 31/32006, or died before 31/3/2008.
Reichard et al., 2012[62]	United States	7/2008 – 6/2009	3,722 (71%)<br><br>18-65	≥1 Measurements during study period: Lipids (any), eye exam.<br><br>*Administrative claims data*	**Primary aim:** To assess Kansas Medicaid data to determine the quality of diabetic care and the level to which individuals with physical disabilities' prevention and diabetes management needs are being met.<br><br>**Data source:** Data extracted from individuals with **physical disabilities and diabetes** who received medical benefits through Kansas Medicaid. Kansas Medicaid program provides insurance coverage for inpatient, outpatient, pharmacy, long term care and hospice coverage to adults with disabilities who qualify for Supplemental Security Income, have high medical needs, qualify for Medicare, or have a severe disability and are awaiting permanent federal disability status. Each of these programs has its own income qualifications. Persons with diabetes-related claims during a 12-month period (7/2007 – 6/2008) were identified and quality of care was followed the subsequent 12 months. All individuals included were continuously eligible for the entire 24 months.
Gold et al., 2012[63]	United States	2005-2007	3,384 (57%)<br><br>Adults	≥3 Measurements during 3-year study period: LDL, microalbuminuria, HbA1c.<br><br>*Electronic medical records*	**Primary aim:** To determine if amount of time with insurance coverage had a dose-response relationship with the likelihood of receiving diabetes preventive care over a three-year study period.<br><br>**Data source:** Electronic health record data extracted from adults with **diabetes** receiving care in 50 safety net clinics in Oregon in 2005–2007. Receipt of these services were assessed using procedure codes associated with each service. Eligible individuals had to have ≥ two diabetes-associated visits over 2004–2005 and also ≥ one visit in 2006 and another in 2007.
Kilbourne et al., 2011[64]	United State	2007	Assumed to be 1,079<br><br>Not specified but assumed to be adults	≥1 Measurements during study period: Eye exam, foot exam.<br><br>*Medical records*	**Primary aim:** To determine whether patients with serious mental illness receiving care in Veterans Affairs mental health programs with collocated general medical clinics were more likely to receive adequate medical care than those in programs without collocated clinics based on a nationally representative sample.<br><br>**Data source:** The study included veteran affairs (VA) patients with diagnoses of **serious mental illness** in fiscal year (FY) 2006–2007 who were also part of the VA's External Peer Review Program (EPRP) FY 2007 random sample and who received care from VA facilities with organizational data from the VA Mental Health Program Survey. EPRP included patient-level chart review quality indicators for common processes of care. Patients were eligible for EPRP chart review if they had an outpatient visit in the immediately preceding month, had an outpatient visit 13–24 months before the chart review month, and did not have a chart review in the preceding three months. Women as well as those with chronic medical conditions, such as **diabetes**, were oversampled.
Stefos et al., 2011[65]	United States	2004	11,211 (NR)<br><br>Adults	Timely eye exam as indicated by disease.<br><br>*Medical records*	**Primary aim:** To assess correlations addressing this central question, namely, how are changes in primary care panel size related to patient processes and satisfaction, and the amount of (waiting) time to be seen by a primary care doctor?<br><br>**Data source:** Patient data from those with **diabetes** extracted from US Department of Veterans Affairs (VA) primary care clinics.VA operates the largest health care system in the US. Data for the analyses on process indicators were gathered from a 2004 sample as part of the External Peer Review Programme.
Fraser et al., 2011[66]	England	7/2010	70,004 (45%)<br><br>≥12	Eye exam within 3 years from study period. (unclear whether all patients where truly referred to a program)<br><br>*Medical records*	**Primary aim:** To compare access and uptake of screening between groups of people with diabetes in each of three screening programs covering this area of southern England.<br><br>**Data source:** Data extracted from a patient-level dataset using data from general practices that refer to three diabetic retinopathy screening programmes and a combined health record. The Hampshire Health Record received data from approximately two thirds of general practices in the region, and from secondary care. It is used by clinicians to share information between primary and secondary care, and provides a rich source of contemporaneous data with potential for public health use. Multiple diabetes diagnosis codes were used in order to capture all registered people with **diabetes**. Diabetic retinopathy screening in England is provided by local programs with guidance and quality assurance oversight from the English National Screening. Programme for Diabetic Retinopathy. Diabetic retinopathy screening is offered annually to all people with diabetes over the age of 12 years.

Williams et al., 2010[67]	United States	2005 (survey year)	2,883 (1,516,171 weighted)<br><br>≥18	≥1 Measurements in the prior 12 months from survey: Feet exam, eye exam, HbA1c.<br><br>*Self-reported*	**Primary aim:** To broaden the examination of diabetes care among patients with mental issues from samples at defined treatment locations to a population-based examination of three aspects of diabetes care among California adults with Type 2 diabetes and serious psychological distress."<br><br>**Data source:** Data extracted from those with **type 2 diabetes** from the 2005 California Health Interview Survey (CHIS), a population-based, random digit dial telephone survey of California households**.** CHIS is the largest state-level survey in the United States, conducted biannually and was designed to provide state-wide approximations for various ethnic groups, with a special effort to include individuals speaking little to no English. Homeless or institutionalized individuals were excluded.
Green et al., 2010[68]	United States	1/2004-12/2006	8,817 (64%)<br><br>18-75	≥1 Measurements in a given year: HDL, LDL, HbA1c, eye exam, nephropathy screening.<br><br>*Administrative claims data*	**Primary aim:** To assess whether practice setting influenced whether patients with mental illness received the same quality of diabetes preventive care as patients without mental illness.<br><br>**Data source:** Data extracted from patients with **diabetes** seen in either the emergency or the outpatient setting of a safety-net health system (large urban public Hospital that serves predominantly uninsured, Medicaid, and Medicare patients), including those with an outpatient or inpatient encounter between 1/2004 and 12/2005. Once enrolled, patients were followed through 12/2006. Patients were included if they had a diabetes diagnosis and a diabetes-related laboratory workup completed in ≥1 of the first two quarters of 2004. To remain in the study, a participant must have had at least two visits, with the last visit ≥6 months later than the first.
Chen et al., 2010[69]	United States	1/1/1999-31/12/2006	Varies per year ranging from 19,573 (48%) in 1999 to 32,365 (47%) in 2006<br><br>18-75	Combination (≥2 HbA1c measurements and ≥1 LDL measurement during 1 year).<br><br>*Administrative claims data*	**Primary aim:** To investigate the effectiveness of a pay-for-performance program to increase the receipt of quality care and to decrease hospitalization rates among patients with diabetes.<br><br>**Data source:** Demographic, pharmacy, inpatient, and outpatient administrative medical claims data from 1/1/1999, through 31/12/2006 were used. The study sample consisted of individuals with **diabetes** who saw Pay for Performance (PP4P)-participating physicians or non–P4P-participating physicians exclusively. Those who saw both P4P-participating and non–P4P-participating physicians were excluded. P4P, implemented by a large provider of healthcare coverage in Hawaii, provides participating physicians with financial incentives to perform quality-of-care processes. Participation in the P4P is voluntary.
Tomio et al., 2010[70]	Japan	5/2006-4/2007	636 (51%)<br><br>NS	≥1 Measurements during study period:HbA1c (≥4), eye exam, nephropathy screening (urinary albumin excretion tests and/or qualitative urine albumin tests, excl. renal patients).<br><br>*Administrative claims data*	**Primary aim:** To assess the quality of diabetes care in two communities in Japan by using National Health Insurance claims data.<br><br>**Data source:** Data extracted from beneficiaries with **diabetes** of National Health Insurance (NHI) in two communities in south-western Japan from 5/2006 to 4/2007. Only those who had ≥1 claim forms with a diagnosis of diabetes mellitus every month from 5/2006 to 4/2007 were included. NHI covers self-employed workers and unemployed. Those with ≥1 claims for hospitalized care claim forms and/or ≥1 diagnosis of disorders in the perinatal period, including gestational diabetes during study period were excluded, as well as those that received non-fee-for service care for at least 1 month.
Wilf-Miron *et al.,* 2010[71]	Israel	12/2007 – 11/2008	74,953 (46%)<br><br>18 - 80	≥1 Measurements during the study period: HbA1c, LDL, combination (HbA1c, LDL, microalbuminuria testing, eye and foot exam, blood pressure, BMI).<br><br>*Administrative data*	**Primary aim:** To explore disparities in diabetes prevalence, care and control among diabetic patients.<br><br>**Data source:** Data extracted from the Maccabi Healthcare Services (MHS), including all MHS members who had visited a general practitioner ≥1 during previous 2 years and were registered as having **diabetes** at 15/11/2008. MHS is an Israeli health plan providing community-based health services throughout the country. Those with gestational diabetes were excluded.
Gregg et al., 2010[72]	United States	1999-2002 (3-years)	8,392 (53%)<br><br>≥18	Combination (NOT receiving HbA1c, cholesterol, albuminuria, eye exam, or foot exam) during study period.<br><br>*Medical record and/or self-reported*	**Primary aim:** To determine the frequency and correlates of persistent long-term gaps in diabetes care.<br><br>**Data source:** Data extracted from patient surveys and reviews of medical records to assess preventive care services for previously diagnosed **type 2 diabetes** among those who were continuously enrolled in 10 US managed care plans from 1999 to 2002. Participants were considered eligible if they had been continuously enrolled in the health plan for at least 3 years, submitted at least 1 claim in the first 18 months, were not pregnant, and participated in follow-up survey. Those with probable type 1 diabetes were excluded. Whether HbA1c, lipid tests, and urine albumin tests were received was based solely on chart abstraction, while eye and foot exam were

					considered to have been received if they were self-reported or recorded in the medical record.
Ng et al., 2010[73]	United States	2004-2006 (Survey period)	4,076 (NR) (13,504,000 (52%) assumed to be weighted)<br><br>≥45	Combination (HbA1c, eye exam and foot exam) in the 12 months prior to survey.<br><br>*Self-reported*	**Primary aim:** To examine the relation of age, gender and insurance status to quality of care among Americans with diabetes and cardiovascular conditions.<br><br>**Data source:** Data extracted from nationally representative MEPS data (2004-2006 pooled). MEPS is a health survey developed to analyze health care use, expenditures and insurance coverage for the U.S. civilian noninstitutionalized population. The MEPS Household Component (MEPS HC) provides estimates of respondents' demographic and socioeconomic characteristics, access to care, health insurance coverage and effectiveness of care for an array of priority clinical conditions, including cardiovascular disease. The MEPS also collects information on diabetes care effectiveness separately through a self-survey, the MEPS Diabetes Care Supplement. Non-institutionalized individuals with self-identified **diabetes** were eligible for inclusion. Older adults who reported being "uninsured" were excluded.
Wang et al., 2010[74]	China	2/2009-11/2009 (data extraction)	824 (59%)<br><br>≥18	≥1 Eye exams within 12 months prior to survey.<br><br>*Medical records or self-reported*	**Primary aim:** To assess the use of eye care and its predictors among diabetic patients in China.<br><br>**Data source:** Between February and November 2009, those with physician-diagnosed **diabetes** were recruited from an urban tertiary and community hospitals and from a rural clinic in Guangdong, China. Subjects having been diagnosed less than 12 months previously or who were unable to cooperate with the interview were excluded. Outcomes were defined according to documentation in the patient's chart, and when this was unavailable or dates were not stated clearly, by the subject's self-report.
Gulliford et al., 2010[75]	England	1/9/2007-28/2/2009	31,484 (49%)<br><br>≥12	<u>No</u> eye exam during study period after invitation.<br><br>*Electronic medical records*	**Primary aim:** To determine the extent of socioeconomic and ethnic differentials in diabetic retinopathy screening uptake and screening outcomes following the implementation of the screening programme.<br><br>**Data source:** Anonymized data extracted from the Diabetes Eye complications service for South East London for all appointments and episodes from 19/2007 to 28/2/2009.The study was set in Lambeth, Southwark and Lewisham. These rank as the 19th, 26th and 39th most deprived local authorities in England. The diabetes retinal screening service in South London is known as the Diabetes Eye Complication Service. There are clinics held on four sites at the three teaching hospitals and one district hospital. Screening is offered to all general practitioner-registered patients over the age of 12 years who have diagnosed **diabete**s. A recall register has been established so that all eligible people with diabetes who are registered with local family practices will automatically be offered appointments.
Lawrenson *et al.,* 2009[76]	New Zealand	15/11/2005 – 15/11/2007	1,111 (49%)<br><br>≥20	Measurements <u>NOT</u> recorded: retinal screening during the last 2 years (excluding newly diagnosed patients).<br><br>*Electronic medical records*	**Primary aim:** To estimate the prevalence of diabetes by age, gender and ethnicity, to look at quality of care, and to investigate disparities in care.<br><br>**Data source:** Data extracted from three general practices in Hamilton (New Zealand), including those **with type 2 diabetes** (prevalent and newly diagnosed).
Guthrie *et al.*, 2009[77]	Scotland	2005/2006	10,161 (47%)<br><br>≥35	≥1 Measurements during previous 12 months: HbA1c, total cholesterol, blood pressure, smoking, combination (all).<br><br>*Electronic medical records*	**Primary aim:** To measure quality of vascular risk factor measurement and control in people with type 2 diabetes after comprehensive pay-for-performance implementation and to examine variation by patient and practice characteristics.<br><br>**Data source:** Data extracted, after pay-for-performance implementation, from the Diabetes Audit and Research in Tayside (Scotland) population diabetes register, including individuals with **type 2 diabetes** on 30/4/2006 diagnosed at ≥35 years.
Gnavi *et al.,* 2009[78]	Italy	1/8/2003 - 31/7/2004	33,453 (49%)<br><br>≥20	≥1 Measurements during study period: HbA1c, cholesterol (total, HDL, and LDL), microalbuminuria, eye exam, combination (HbA1c and ≥2 assessments from among eye exam, total cholesterol and microalbuminuria).<br><br>*Administrative claims data*	**Primary aim:** To investigate the role of clinical and socioeconomic variables as determinants of adherence to recommended diabetes care guidelines and assess disparities in the process of care between diabetologists and general practitioners.<br><br>**Data source:** All residents in Torino (Italy) with a diagnosis of **diabetes** and being alive at 31/7/2003 were eligible for inclusion. All laboratory tests and specialist medical examinations reimbursed by the national health service in the study period were linked to the population with diabetes to identify process of care.

Kirkbride et al., 2009[79]	United States	2002 and 2003	6,267 (65%)<br><br>18-64	≥1 Measurements during the calendar year: HbA1c, lipid profile, eye exam.<br><br>*Administrative claims data*	**Primary aim:** To assess whether Rural Health Clinics were associated with higher rates of recommended primary care services for adult beneficiaries diagnosed with diabetes in Oregon's Medicaid program, the Oregon Health Plan.<br><br>**Data source:** Data extracted from Oregon's Medicaid program, the Oregon Health Plan from 2002 to 2003 to assess quality of diabetic care for beneficiaries with **diabetes** residing in urban areas or rural areas with or without at least 1 rural health clinic. Study subjects included Temporary Assistance to Needy Families or disabled beneficiaries who were enrolled in the health plan for 12 months per study year and had at least 1 claim with a diabetes diagnosis. Those with gestational diabetes and those who gave birth during a given study year were excluded, as well as those in areas where rural health clinic was new in that year.

Study details can be found in the original articles.

Supplemental table III. Studies excluded from the qualitative analyses because of overlapping patient populations or because studies were repeated over time.

**First author, year**	**(Partial) overlap with/ more recent data available from**	**Outcomes not included in qualitative analyses**<br><br>*OR (95% CI), ref = men, unless otherwise specified*	**Level of adjustment**
Peraj et al., 2019[80] *(Fully excluded)*	Kamat et al., 2019	Foot exam prior 12 months: 0.91 (0.67, 1.25)	Multivariable
Barker et al., 2018[81] *(Fully excluded)*	Kiran et al., 2012 *Less recent but larger study population not restricted to those with mental illness.*	≥1 Measurements during study period:<br>Eye exam: 1.13 (1.08;1.19)<br>HbA1c (≥4): 1.06 (1.01;1.12)<br>Dyslipidemia: 1.04 (0.99;1.11)<br>HbA1c: 1.20 (1.10;1.30)<br>Combination (≥1 of the above):1.16 (1.08;1.24)	Multivariable
Canedo et al., 2018[82] *(Fully excluded)*	Comer-HaGans et al., 2020 and Bennet et al., 2017	HbA1c (≥2) prior 12 months: 1.14 (0.82;1.58)<br>Foot exam prior 12 months: 0.95 (0.72;1.26)<br>Eye exam prior 12 months:1.14 (0.87;1.47)<br>Cholesterol prior 12 months: 1.03 (0.76;1.41)	Multivariable
Williams et al., 2017[28] *(Partially excluded)*	Comer-HaGans et al., 2020	HbA1c (≥2) prior 12 months: 1.01 (0.89;1.14)<br>Eye exam prior 12 months: 1.14 (1.04;1.24)<br>Foot exam prior 12 months: 0.91 (0.83;1.00)	Multivariable
National Diabetes Audit 2016-2017[22] *(Partially excluded)*	National Diabetes Audit 2018-2017	≥1 measurements during study period<br>HbA1c: 1.12 (1.11;1.14)<br>Blood pressure: 1.16 (1.14;1.17)<br>Cholesterol:0.97 (0.96;0.98)<br>Urine albumin: .89 (0.88;0.89)<br>Smoking: 87 (0.87;0.88)<br>Combination: 0.92 (0.91;0.92)	Multivariable
National Diabetes Audit 2015-2016[22] *(Fully excluded)*	National Diabetes Audit 2018-2017	≥1 measurements during study period<br>Urine albumin: 0.90 (0.89;0.91)<br>Foot exam: 0.99 (0.98;1.00)<br>BMI: 0.98 (0.97;0.99)<br>Smoking: 0.86 (0.85;0.86)<br>Combination: 0.91 (0.90;0.91)	Multivariable
National Diabetes Audit 2014-2015[22] *(Fully excluded)*	National Diabetes Audit 2018-2017	≥1 measurements during study period<br>Blood pressure: 1.12 (1.10;1.13)<br>Cholesterol: 0.98 (0.97;0.99)<br>Urine albumin: 0.93 (0.92;0.94)<br>Foot exam: 0.99 (0.98;1.00)<br>BMI: 0.98 (0.97;0.99)<br>Smoking: 0.87 (0.86;0.88)<br>Combination: 0.94 (0.93;0.95)	Multivariable
National Diabetes Audit 2014-2013[22] *(Fully excluded)*	National Diabetes Audit 2018-2017	≥1 measurements during study period<br>Urine albumin: 0.93 (0.92;0.94)<br>Smoking: 0.86 (0.85;0.87)<br>Combination: 0.93 (0.92;0.94)	Multivariable
National Diabetes Audit 2013-2012[22] *(Partially excluded)*	National Diabetes Audit 2018-2017	≥1 measurements during study period<br>HbA1c: 1.01 (1.00;1.03)<br>Blood pressure: 1.14 (1.12;1.16)<br>Cholesterol: 0.93 (0.92;0.94)<br>Urine albumin: 0.85 (0.85;0.86)<br>Foot exam: 0.97 (0.97;0.98)<br>BMI: 0.92 (0.91;0.93)	Multivariable

		Smoking: 0.87 (0.86;0.88)<br>Combination:0.85 (0.85;0.86)	
National Diabetes Audit 2012-2011[22]<br>*(Fully excluded)*	National Diabetes Audit 2018-2017	≥1 measurements during study period<br>HbA1c: 1.04 (1.03;1.05)<br>Blood pressure: 1.14 (1.13;1.16)<br>Cholesterol: 0.95 (0.94;0.96)<br>Creatinine: 1.04 (1.03;1.05)<br>Urine albumin: 0.89(0.88;0.89)<br>Foot exam: 0.98 (0.98;0.99)<br>BMI: 0.92 (0.91;0.93)<br>Smoking: 0.89 (0.88;0.89)<br>Combination: 0.88 (0.88;0.89)	Multivariable
Bennet et al., 2017[27]<br>*(Partially excluded*)	Comer-HaGans et al., 2020	Eye exam prior 12 months: 1.01 (0.92;1.10)<br>Foot exam prior 12 months: 0.85 (0.78;0.92)<br>HbA1c (≥2) prior 12 months: 0.86 (0.79;0.95)	Multivariable
Sieng et al., 2017[83]<br>*(Fully excluded)*	Sieng et al., 2015[38]	Eye exam prior 12 months: 1.20 (1.12–1.29)<br>Foot exam prior 12 months: 1.12 (1.04–1.21)<br>Combination (LDL, foot exam, eye exam, HbA1c (≥2)) prior 12 months: 1.11 (1.03–1.21)	Multivariable
Doucette et al., 2017[84]<br>*(Fully excluded)*	Chen et al., 2014<br>*Less recent but larger study population*	HbA1c (≥2) prior 12 months: 1.07 (0.89, 1.29)<br>Foot prior 12 months: 1.00 (0.83, 1.21)<br>Eye exam prior 12 months: 1.05 (0.88, 1.25)	Multivariable
Storey et al., 2016[85]<br>*(Fully excluded)*	Murchinson et al., 2017[30]	Follow-up eye exam <15 months for mild, <12 months for moderate diabetic retinopathy and <4 months from the index visit for severe diabetic retinopathy: 0.83 (0.68;1.02)	Multivariable
Sohn et al., 2016[86]<br>*(Fully excluded)*	Chen et al., 2014[52]	Eye exam prior 12 months: 1.07 (1.00;1.15)<br>Foot exam prior 12 months: 0.90 (0.84;0.96)<br>≥2 HbA1c prior 12 months: 1.09 (1.02;1.16)	Multivariable
Mahmoudi et al., 2016[87]<br>*(Fully excluded)*	Comer-HaGans et al., 2020	Eye exam prior 12 months: 1.03 (0.81;1.25)<br>Foot exam prior 12 months: 0.78 (0.62;0.94)<br>Cholesterol prior 12 months: 1.25 (0.86;1.64)	Multivariable
Doucette et al., 2016[88]<br>*(Fully excluded)*	Kamat et al., 2019[16]	Eye exam prior 12 months: 1.69 (0.94;3.03)<br>Foot exam prior 12 months: 1.30 (0.82;2.08)	Multivariable
Shi et al., 2014[89]<br>*(Fully excluded)*	Comer-HaGans et al., 2020	Eye exam prior 12 months per survey year:<br>2002: 0.92 (0.69;1.22)<br>2003: 0.70 (0.51;0.98)<br>2004: 0.95 (0.68;1.32)<br>2005: 0.91 (0.65;1.27<br>2006: 0.83 (0.63;1.08)<br>2007: 0.85 (0.65;1.10)<br>2008: 0.71 (0.53;0.94)<br>2009: 0.82 (0.64;1.05)	Multivariable
Hu et al., 2014[90]<br>*(Fully excluded)*	Comer-HaGans et al., 2020 and Bennet et al., 2017	Eye exam prior 12 months: 1.35 (1.07;1.70)<br>Foot exam prior 12 months: 0.83 (0.63;1.10)<br>Cholesterol prior 12 months: 1.21 (0.91;1.61)<br>HbA1c prior 12 months: 1.31 (0.84;2.04)	Multivariable
Chou et al., 2012[91]<br>*(Fully excluded)*	Chen et al., 2014[52]	Eye exam prior 12 months: 1.16 (1.03;1.30)	Multivariable
Hale et al., 2010[92]<br>*(Fully excluded)*	Chen et al., 2014[52]	Eye exam prior 12 months: 1.12 (0.96;1.30)<br>Foot exam prior 12 months: 0.86 (0.75; 1.00)<br>≥2 HbA1c prior 12 months: 1.18 (1.01;1.35)	Multivariable
Byun et al., 2013[93]	Rim et al., 2013	Eye exam prior 12 months: 1.19 (0.88;1.62)	Multivariable

(Fully excluded)			
Richard et al., 2012[94] *(Fully excluded)*	Comer-HaGans et al., 2020	HbA1c prior 12 months: 1.20 (0.93;1.47)<br>Eye exam prior 12 months: 1.07 (0.88;1.26)<br>Foot exam prior 12 months: 0.91 (0.72;1.11)	Multivariable
Richard et al., 2011[95] *(Fully excluded)*	Comer-HaGans et al., 2020	Eye exam prior 12 months: 1.14 (0.93;1.40)<br>Foot exam prior 12 months: 1.10 (0.90;1.35)<br>HbA1c (≥2) prior 12 months: 1.14 (0.96;1.35)	Multivariable
Do et al., 2011[96] *(Fully excluded)*	Rim et al., 2013	Eye exam prior 12 months: 1.59 (1.21;2.07)<br>Microalbuminuria prior 12 months: 1.34 (1.04;1.72)	Multivariable
Ng et al., 2010[73] *(Partially excluded)*	Comer-HaGans et al., 2020 and Williams et al., 2017	HbA1c in prior 12 months: 1.26 (0.95;1.67)<br>Blood pressure in prior 12 months: 1.65 (0.93;2.94)<br>Cholesterol in prior 24 months: 1.44 (0.95;2.18)<br>Eye exam in prior 12 months: 1.10 (0.94;1.30)<br>Foot exam in prior 12 months: 0.97 (0.80;1.17)<br>Pooled data	Multivariable

OR = odds ratio; CI = confidence interval.

Supplemental table IV. Studies only presenting unadjusted data.

First author,<br>year	Country	Study period	Study size<br>(% women)	Outcome<br>*OR (95% CI), ref = men,*	
Backe et al., 2020[97]	Greenland	30/11/2018<br>(data extraction)	1,498 (48%)	HbA1c<br>Blood pressure<br>Microalbuminuria<br>Eye exam<br>Foot exam	*1.48 (1.08;2.03)±*<br>*1.55 (1.20;2.01)±*<br>1.00 (0.81;1.25)±<br>1.10 (0.86;1.42)±<br>0.99 (0.81;1.22)±
Boucher et al., 2020[98]^	Canada	3/2018-6/2018<br>(Survey period)	148 (45%)	Eye exam	0.64 (0.20;2.08)±
Benoit et al., 2019[99]	United States	2010-2014	355,384<br>(52%)	Eye exam	*1.05 (1.03;1.07)±*
Gediminas et al., 2019[100]	Lithuania	2011	382 (61%)	BMI<br>Foot exam<br>Eye exam<br>HbA1c<br>LDL<br>Creatinine<br>Blood pressure	1.0 (0.6-1.6)<br>1.3 (0.8-2.2)<br>*1.6 (1.1-2.4)*<br>1.4 (0.9-2.1)<br>1.3 (0.7-2.2)<br>1.0 (0.7-1.6)<br>-
Wright *et al.*, 2019[101]	England	2006-2013	Presented by years since diagnosis: 4,221 (46%) to 30,501 (43%)	Years 2-3<br>HbA1c<br>Blood pressure<br>Microalbuminuria<br>eGFR or creatinine<br>BMI<br><br>Years 4-5<br>HbA1c<br>Blood pressure<br>Microalbuminuria<br>eGFR or creatinine<br>BMI<br><br>Years 6-7<br>HbA1c<br>Blood pressure<br>Microalbuminuria<br>eGFR or creatinine<br>BMI	 1.02 (0.92;1.13)<br>*1.15 (1.03;1.30)*<br>*0.88 (0.84;0.92)*<br>*1.20 (1.08;1.33)*<br>0.98 (0.90;1.06)<br><br><br>0.98 (0.85;1.14)<br>1.15 (0.97;1.35)<br>*0.88 (0.82;0.94)*<br>1.04 (0.89;1.20)<br>0.98 (0.87;1.10)<br><br><br>0.84 (0.63;1.12)<br>0.81 (0.60;1.08)<br>*0.82 (0.72;0.93)*<br>0.85 (0.64;1.14)<br>*0.80 (0.65;0.99)*
Nazu et al., 2019[102]	Finland	2011-2016	8,429 (47%)	2015-2016<br>HbA1c<br>LDL	 *1.35 (1.18;1.54)±*<br>0.93 (0.82;1.04)±
Corrao et al., 2019[103]	Italy	2010 (year of diagnosis)	77,285<br>(47.5%)	Combination	*0.85 (0.82;0.88))±*
Tracey et al., 2019[104]^	Ireland	11/2013-8/2015<br>(data extraction)	582 (39%)	Eye exam:	*0.33 (0.12;0.92)±*
Mesa et al., 2018[105]	Unites States	2015	100 (50%)	HbA1c<br>LDL<br>Eye exam	0.74 (0.30;1.79)±<br>1.71 (0.52;5.66)±<br>0.71 (0.31;1.60)±
Al-Salameh et al., 2018[106]	France	4/2009 – 6//2014<br>(inclusion period: 4/2009 – 6/2011)	983 (47%)	Lipid profile	0.96 (0.65;1.42)±
Bird *et al.*, 2018b[107]	Unites Stated	2011 and 2012	Varies per outcome of interest	LDL<br>HbA1c<br>Eye exam<br>Renal test	*1.09 (1.07;1.12)*<br>*1.19 (1.16;1.22)*<br>*1.28 (1.26;1.30)*<br>*1.04 (1.01;1.06)*
Diabetic Retina-Screen 2013-2015[108]^	Ireland	2013-2014	69,894 (41%)	Eye exam year 1	*0.77 (0.74;0.81)±*

		2015	88,668 (41%)	Eye exam year 2	*0.84 (0.81;0.88)±*
Statistical Bulletin 2016-2017[109]^	Ireland	2016	105,915 (41%)	Eye exam year 3	*0.86 (0.83;0.89)±*
		2017	114,078 (41%)	Eye exam year 4	*0.83 (0.80;0.86)±*
Kekäläinen et al., 2016[110]	Finland	2013-2014	1,075 (41%)	HbA1c<br>LDL	*2.24 (1.32;3.82)±*<br>*2.12 (1.36;3.33)±*
Han et al., 2016[111]	Korea	2013 (survey year)	20,806 (52%)	Combination	*0.89 (0.84;0.94)±*
Ferroni et al., 2016[112]	Italy	2013	139,935 (43%)	HbA1c<br>Microalbuminuria<br>Lipid profile	*1.04 (1.02;1.07)±*<br>*0.94 (0.92;0.96)±*<br>1.01 (0.99;1.04)±
Cambra et al., 2016[113]	Spain	15/5/2014 (index date)	32,220 (44%)	HbA1c<br>Blood pressure<br>LDL<br>HDL<br>Triglycerides<br>BMI<br>Smoking	1.03 (0.99;1.09)±<br>*1.30 (1.24;1.37)±*<br>*1.09 (1.04;1.15)±*<br>*1.06 (1.01;1.12)±*<br>*1.06 (1.01;1.12)±*<br>1.02 (0.97;1.06)±<br>0.91 (0.87;0.96)±
Seghieri et al., 2016[114]	Italy	2006	91,826 (49.7%)	Urine albumin<br>HbA1c<br>Eye exam<br>Lipid profile<br>Combination	*0.93 (0.91;0.97)±*<br>*1.08 (1.06;1.11)±*<br>*1.09 (1.06;1.12)±*<br>*1.08 (1.05;1.10)±*<br>*1.04 (1.01;1.07)±*
Cleland et al., 2016[115]^	Tanzania	2011-2014	5,729 (60%)	Eye exam	*1.36 (1.22;1.52)*
Manicardi *et al.,* 2016[116]	Italy	2011	28,802 (46%)	HbA1c<br>lipid profile<br>Blood pressure<br>Renal function<br>Eye exam	1.03 (0.94;1.14)±<br>1.01 (0.96;1.07)±<br>1.03 (0.97;1.09)±<br>1.02 (0.98;1.07)±<br>1.01 (0.97;1.06)±
Hwang et al., 2016[117]	Korea	2005, 2007-2009	2,214 (53%)	Eye exam	1.15 (0.97;1.36)
Keenum et al., 2016[118]^	United States	26/1/2012-1/5/2015	949 (65%)	Eye exam	1.16 (0.87;1.56)±
Szabo et al., 2015[119]	United Arab Emirates	2010	150 (69%)	HbA1c<br>LDL<br>Eye<br>Renal exam<br>Combination	-<br>2.83 (0.90;8.94)±<br>0.57 (0.27;1.19)±<br>0.53 (0.24;1.19)±<br>1.26 (0.63;2.52)±
Afandi et al., 2015[120]	United Arab Emirates	2013	240 (58%)	BMI	100%/100%
Hendriks et al., 2015[121]	The Netherlands	2013	42,641 (46%)	HbA1c<br>Systolic BP<br>Smoking<br>TC/HDL-ratio<br>ACR<br>Foot exam<br>Eye exam<br>BMI	*1.10 (1.00;1.21)±*<br>1.07 (0.96;1.19)±<br>*1.15 (1.04;1.28)±*<br>*1.12 (1.02;1.23)±*<br>*0.93 (0.88;0.98)±*<br>*1.09 (1.03;1.15)±*<br>1.03 (0.98;1.09)±<br>*1.10 (1.00;1.20)±*
Ballotari *et al.,* 2015[122]	Italy	2010	16,903 (42%)	HbA1c	*1.10 (1.03;1.18)±*
Russo et al., 2015[123]	Italy	2009	415.294 (45%)	Lipid profile	0.91 (0.90;0.93)±
Onakpoya et al., 2015[124]^	Nigeria	7/2010-11/2010 (inclusion period)	179 (49%)	Eye exam	0.71 (0.39;1.28)±

Kiran et al., 2014[125]	Canada	2006-2008	734,739 (48%)	Eye exam<br>HbA1c<br>Cholesterol<br>Combination	*1.15 (1.14;1.16)±*<br>1.00 (0.99;1.01)±<br>*0.93 (0.92;0.94)±*<br>*1.03 (1.02;1.04) ±*
Bayer et al., 2014[126]	United States	2003	1,797 (17%)	Combination	0.79 (0.55;1.14)±
Chou et al., 2014[127]	United States	2006-2010 (survey period)	27,699 (NR)	Eye exam	P-value<br>0.089
Matheka et al., 2013[128]	Kenya	10/2012-11/2012 (survey period)	198 (70%)	HbA1c	0.33 (0.16;0.67)±
Kautzky-Willer et al., 2013	Austria	3/2009-8/2009 (data collection)	225 (45%)	HbA1c	0.82 (0.31;2.14)±
Kiran et al., 2013[129]	Canada	2010	851,193 (48%)	Eye exam	*1.15 (1.14;1.16)±*
Cetin et al., 2013[130]	Turkey	1/2010-5/2010 (survey period)	437 (52%)	Eye exam	0.81 (0.51;1.28)±
Paksin et al., 2013[131]	United States	2009 (survey year)	52,386 (59%) (49% weighted)	Eye exam	*p-value*<br>*0.641*
Driskell et al., 2012[132]	England	2010	54 537 (47%)	HbA1c	*0.90 (0.86;0.93)±*
Orton et al., 2013[133]^	England	1/2009-7/2010 (screening invitation period)	47,111 (44%)	Eye exam	1.04 (0.99;1.08)
Sachdeva et al., 2012[134]^	England	2008	611 (47%)	Eye exam	1.24 (0.89;1.72)±
Arcury et al., 2012[135]	United States	6/2009-2/2010 (data collection)	563 (62%)	HbA1c<br>Feet exam	1.04 (0.61;1.78)±<br>1.37 (0.90;2.08)±
Van Eijk et al., 2012[136]	The Netherlands	2008 (questionnaire)	1,891 (51%)	Eye exam	1.00 (0.78;1.28)±
Wong et al., 2012[137] *Multivariable analyses but not for age and therefore excluded from qualitative analyses*	China	2008 - 2009	1,970 (55%)<br><br>NS	HbA1c<br>Cholesterol<br>Smoking<br>Microalbuminuria<br>Eye exam<br>BMI	0.84 (0.58;1.20)<br>0.92 (0.66;1.28)<br>*0.61 (0.43;0.87)*<br>0.83 (0.67;1.03)<br>1.13 (0.93;1.38)<br>0.95 (0.75;1.21)
Sundquist et al., 2011[138]	Sweden	2005	5,048 (42%)	HbA1c<br>Lipids	*1.27 (1.03;1.56)±*<br>*1.30 (1.13;1.50)±*
Sadowski et al., 2011[139]	United States	9/2009-12-2009 (data collection)	134 (59%)	HbA1c<br>Foot exam<br>Eye exam<br>Cholesterol<br>Combination	1.73 (0.74;4.05)±<br>1.39 (0.63;3.05)±<br>0.45 (0.19;1.06)±<br>0.32 (0.03;2.97)±<br>1.07 (0.54;2.14)±
De Lusignan et al., 2011[140]	England	2007	6,897 (47%)	Creatinine<br>Microalbuminuria<br>Macroalbuminuria	1.18 (0.92;1.50)±<br>0.91 (0.81;1.03)±<br>0.99 (0.87;1.11)±
Morren et al., 2011[141]	Caribbean	28/10/2007-29/11/2007 (patient interviews)	225 (65%)	Total cholesterol<br>HbA1c	*2.14 (1.20;3.82)±*<br>*2.19 (1.24;3.87)±*
Onakpoya et al., 2010[142]	Nigeria	11/2007	83 (61%)	Eye exam	0.94 (0.35;2.50)±
Goh et al., 2010[143]	Malaysia	2006	2,373 (57%)	Eye exam	0.94 (0.75;1.19)±
Gossain et al., 2010[144]	United States	1/2006-6/2008 (data extraction)	499 (52%)	HDL year 1<br>HDL year 2<br>Blood pressure	1.10 (0.57;2.09)±<br>1.05 (0.66;1.68)±<br>-

Shireman et al., 2010[145]	United States	9/2006-8/2007	666 (50%)	Lipids<br>Microalbuminuria<br>Eye exam	0.89 (0.65;1.20)±<br>1.30 (0.88;1.92)±<br>1.01 (0.73;1.42)±
Banta et al., 2009[146]	United States	5/2004-4/2005	482 (68%)	HbA1c<br>Lipid<br>Eye exam	1.21 (0.82;1.78)±<br>*1.60 (1.09;2.36)±*<br>1.33 (0.87;2.03)±
Fischbacher et al., 2009[147]	Scotland	11/2003-12/2004	9,833 (47%)	HbA1c<br>Cholesterol<br>Blood pressure<br>Eye exam<br>BMI	0.90 (0.73;1.10)±<br>0.86 (0.73;1.01)±<br>0.97 (0.85;1.11)±<br>*0.88 (0.79;0.99)±*<br>0.92 (0.82;1.04)±

If studies presented sex-specific numbers and percentages without reporting a measure of association, crude odds ratios (ORs) and 95% confidence intervals (CIs) were calculated using Review Manager 5.3. ^ = Eye exam attendance after invitation

Supplemental table IV. A Modified Newcastle-Ottawa quality assessment scale to assess risk of bias.

	**Selection (out of 3)**			**Comparability (out of 2)**		**Outcome (out of 1)**	
First name, year	*Representativeness of the exposed cohort*	*Selection of the non-exposed cohort*	*Ascertainment of exposure (= sex)*	*Study controls for one variable*	*Study controls for any additional variable(s)*	*Assessment of outcome*	
Swietek et al., 2020	0 (specific geographical area (North Carolina, Georgia, and Texas, US), diabetes + depressive disorders, Medicaid enrolee, working age adults	*	* (administrative data)	*	*	* (administrative data)	Fair
Lu et al., 2020	0 (restricted to intellectual and developmental disabilities and diabetes or diabetes only in specific geographical areas (Iowa, Massachusetts, New York, Oregon and South Carolina, US), Medicaid enrolee, working age adults	*	* (administrative claims data)	*	*	* (administrative claims data)	Fair
Wei et al., 2020	0 (restricted to those receiving glucose-lowering medication, enrolees of a specific insurance company (Switzerland))	*	* (administrative claims data)	*	*	* (administrative claims data)	Fair
Youn et al., 2020	* (nationwide survey (Korea))	*	* (self-reported through trained interviewers)	*	*	0 (self-reported (trained interviewers))	Good
Tan et al., 2020	*/0 (stratified random sample (US), type 2 DM, had at least one clinical measurement))	*	* (self-reported through self-administered internet-based questionnaire)	*	*	* (combination of health records and self-reported including sensitivity analysis)	Fair
Meier et al., 2020	0 (electronic medical records database of the Institute of Primary Care of the University of Zurich.	*	* (electronic medical records)	*	*	* (electronic medical records)	Fair
Comer-Hagans et al., 2020	* (population-based (MEPS, US)	*	* (self-reported)	*	*	0 (self-reported)	Poor
Hirst et al., 2019	0 (only those with a minimum number of HbA1c tests post diagnosis, primary care (UK))	*	* (primary care medical record database)	*	*	* (primary care medical record database)	Fair
Bakke et al., 2019	* (population-based (Norway), primary care, type 2 diabetes)	*	* (primary care medical records)	*	*	* (primary care medical records)	Good
Dallo et al., 2019	*/0 (racially diverse population, restricted to metropolitan Detroit (US))	*	* (medical records)	*	*	* (medical records)	Fair
De Jong et al., 2019	*/0 (population-based, one geographical region (Utrecht, The Netherlands), primary care)	*	* (primary care medical records)	* (age)	0	* (primary care medical records)	Fair
Whyte et al., 2019	* (population-based (England), type 2 diabetes, primary care)	*	* (primary care medical records)	*	*	* (primary care medical records)	Good
Du et al., 2019	*/0 (national representative sample (Germany), type 2 diabetes, relatively small sample)	*	* (self-report through computer-assisted interview)	*	*	0 (self-report through computer-assisted interview)	Poor

Kovács et al., 2019	*/0 (population-based (Hungary), restricted to those receiving glucose-lowering medication)	*	* (primary care medical records)	*	*	* (primary care medical records)	Fair
Kamat et al., 2019	* (Population-based, complex, stratified, multistage, probability sampling design (NHANES, US))	*	* (Self-reported through interview)	*	*	0 (Self-reported through interview)	Poor
An et al., 2018	*/0 (only those in Southern California (US), restricted to those with two or more outpatient visits)	*	* (medical records)	*	*	* (medical records)	Fair
Ibáñez et al., 2018	*/0 (population-based, specific geographical area (Navarre, Spain), type 2 diabetes)	*	* (primary care medical records)	*	*	* (primary care medical records)	Fair
Bird et al., 2018a	0 (four metropolitan areas (Atlanta, Georgia; Houston, Texas; New York City/Northern New Jersey; and Southern California, US), commercial health plan members)	*	* (administrative data)	*	*	* (administrative data)	Fair
Kreft et al., 2018	0 (aged 50+, incident diabetes, member of a large insurance provider (Germany))	*	* (Administrative claims data)	*	*	* (Administrative claims data)	Fair
Kawamura et al., 2018	*/0 (only those with incident type 2 diabetes using oral glucose-lowering drugs (Japan))	*	* (Administrative claims data)	*	*	* (Administrative claims data)	Fair
National diabetes Audit	* (population-based (England and Wales))	*	* (Medical records)	*	*	* (Medical records)	Good
Foreman et al., 2017	* (random clustering sampling approach across 30 geographical sites (Australia), aged 40+/50+)	*	* (self-reported through interview)	*	*	0 (self-reported through interview)	Poor
Mwangi et al., 2017	0 (living in Kenya, attending the clinic, random sample, small sample size)	*	0 (self-reported through interview)	*	*	0 (self-reported through interview)	Poor
LeBlanc et al., 2017	*/0 (followed by family physicians paid by fee-for-service, specific region (Canada))	*	* (Administrative data)	*	*	* (Administrative data)	Fair
Yoo et al., 2017	0/* (population-based, restricted to those receiving glucose-lowering medication, more than one claim for diabetes over the year (Korea))	*	* (Administrative claims data)	*	*	* (Administrative claims data)	Fair
Bennet et al., 2017	* (population-based (US, MEPS))	*	* (self-reported through computer-assisted interview)	*	*	0 (Self-reported through computer-assisted interview)	Poor
Williams et al., 2017	* (population-based (US, MEPS))	*	0 (Self-reported through computer-assisted interview)	*	*	0 (Self-reported through computer-assisted interview)	Poor
Willis et al., 2017	0 (one geographical area (West Yorkshire. England), type 2 diabetes)	*	* (primary care medical records)	*	*	* (primary care medical records)	Fair
Moreton et al., 2017	0 (those (newly) referred to a specific screening program, one geographical area (Oxfordshire, England))	*	* (electronic records)	*	*	* (electronic records)	Fair
Murchison et al., 2017	0 (Only those included that received a previous eye exam during follow-up at an urban clinic (US))	*	* (billing and administrative data)	*	*	* (billing and administrative data)	Fair
Tanaka et al., 2016	0 (only those with frequent visits in the prior year and visiting the clinic during study	*	* (administrative claims data)	*	*	* (administrative claims data)	Fair

	period, beneficiaries covered by Health Insurance Societies, type 2 diabetes (Japan))						
Mtuya et al., 2016	0 (specific geographical area (Kilimanjaro Region, Tanzania), only those referred after screening for retinopathy)	*	* (self-reported through interview)	*	*	0 (self-reported through interview)	Poor
Rossaneis et al., 2016	0 (urban area of a large city in the South of Brazil, type 2 diabetes, aged 40+)	*	* (assumed to be self-reported through interview)	*	*	0 (assumed to be self-reported through interview)	Poor
Tannenbaum et al., 2016	0 (specific study location (HCHS/SOL Miami site, US), Hispanics/Latinos, aged 40+)	*	* (self-reported)	*	*	0 (self-reported)	Poor
Hatef et al., 2015	0 (Medicaid patients covered by Johns Hopkins HealthCare), working age adults)	*	* (Administrative claims data)	*	*	* (Administrative claims data)	Fair
Baumeister et al., 2015	*/0 (population-based, a specific geographical area (West Pomerania, Germany))	*	* (Self-reported)	*	*	0 (Self-reported)	Poor
Sieng et al., 2015	*/0 (from all provinces in Thailand, type 2 diabetes, data extracted from those attending the clinic in a given period)	*	* (medical records)	*	*	* (medical records)	Fair
Mounce et al., 2015	*/0 (population-based (England), 50+)	*	* (self-reported, through interview)	*	*	0 (self-reported, through interview)	Poor
Liang et al., 2015	*/0 (population-based, type 2 diabetes,40+, using glucose-lowering medication (UK))	*	* (medical records)	*	*	* (medical records)	Fair
Hwang et al., 2015	* (population-based, type 2 diabetes)	*	* (self-reported through computer assisted telephone interviewing)	*	*	0 (Self-reported through computer assisted telephone interviewing)	Poor
Casanova et al., 2015	0 (specific geographical area (PACA, France), glucose-lowering medication, type 2 diabetes, regional health insurance)	*	* (administrative claims data)	*	*	* (administrative claims data)	Fair
Devkota et al., 2015	0 (only those attending residency clinics, type 2 diabetes, small study size)	*	* (medical records)	*	*	* (Medical records)	Fair
Billimek et al., 2015	*/0 (type 2 diabetes, and encounter with a doctor in previous 12 months, assumed to be in a specific geographical area (California))	*	* (medical records)	*	*	* (medical records	Fair
Al-Sayah et al., 2015	*/0 (type 2 diabetes, specific geographical area (Alberta, Canada)	*	* (self-reported)	*	*	* (self-reported)	Fair
Van Doorn-Klomberg et al., 2015	* (population-based (The Netherlands)	*	* (primary care medical records)	*	*	* (primary care medical records)	Good
Lee et al., 2014	0 (only those visiting a specific health care centre (US), only those without diabetic complications)	*	* (medical records)	*	*	* (medical records + self-report among those without eye exam reported in medical records)	Fair
MacLennan et al., 2014	0 (those visiting an internal medicine clinic of a large, urban, county hospital that serves primarily low income, non-Hispanic African American patients)	*	* (medical records, billing data)	*	*	* (medical records)	Fair

Buja et al., 2014	* (six regions in Italy)	*	* (administrative data)	*	*	* (administrative data)	Good
Naicker et al., 2014	*/0 (specific geographical area (Eastern Ontario, Canada), aged 40+, only practices included that were willing to participate in an improvement initiative)	*	* (medical records)	*	*	* (medical records)	Fair
Baviera et al., 2014	*/0 (specific geographical area (Lombardy, Italy), aged 40+)	*	* (administrative data)	*	*	* (administrative data)	Fair
Chen et al., 2014	* (population-based (BRFSS, US)	*	* (self-reported through telephone survey)	*	*	0 (self-reported through telephone survey)	Poor
Rim et al., 2013	* (population-based (KNAHES, Korea)	*	* (self-reported)	*	*	0 (self-reported)	Poor
Yu et al., 2013	0 (specific geographical area (Washington and Idaho, US), patients from 9 primary care practices that responded to the survey)	*	* (assumed to be self-reported)	*	*	* (self-reported + medical records)	Fair
Rossi et al., 2013	* (population-based (Italy), those referred to the participating outpatient clinics in 2009	*	* (medical records)	*	*	* (medical records)	Fair
Hellemons et al., 2013	*/0 (specific geographical area (Groningen, The Netherlands), type 2 diabetes)	*	* (primary care medical records)	*	*	* (primary care medical records)	Fair
Mier et al., 2012	0 (Hispanics living in Hidalgo County, Texas, at the Texas–Mexico border (US))	*	* (self-reported through interview)	*	*	0 (self-reported through interview)	Poor
Druss et al., 2012	0 (only those with Medicaid fee-for-service, diabetes + mental comorbidity, aged below 65)	*	* (claims data)	*	*	* (claims data)	Fair
Bartels et al., 2012	0 (national sample of Medicare beneficiaries (US), aged 65+)	*	* (claims data)	*	*	* (claims data)	Fair
Chien et al., 2012	0 (those enrolled in a not-for-profit Medicaid-focused managed care plan, specific geographical area (Hudson valley region of New York (US))	*	* (administrative data)	*	*	* (administrative data)	Fair
Kiran et al., 2012	*/0 (specific geographical area (Ontario, Canada), aged 40+)	*	* (administrative claims data)	*	*	* (administrative claims data)	Fair
Reichard et al., 2012	0 (Kansas Medicaid beneficiaries (US), working age adults, diabetes + physical disabilities)	*	* (administrative claims data)	*	*	* (administrative claims data)	Fair
Gold et al., 2012	0 (those receiving care at safety net clinic in a specific geographical area (Oregon, US), minimum number of diabetes-associated visits during study period)	*	* (electronic medical records)	*	*	* (electronic medical records)	Fair
Kilbourne et al., 2011	0 (those receiving care in Veterans Affairs mental health programs, diabetes + mental illness (US), sample size unclear)	*	* (medical records)	*	*	* (medical records)	Fair
Stefos et al., 2011	0 (those seen by Veterans Affairs primary care clinics (US)	*	* (medical records)	*	*	* (medical records)	Fair
Fraser et al., 2011	0 (those being invited for eye screening, specific geographical region (Hampshire, England))	*	* (medical records)	*	*	* (medical records)	Fair

Williams et al., 2010	*/0<br>(population-based, specific geographical area (California, US), type 2 diabetes)	*	*<br>(self-reported through telephone survey)	*	*	0<br>(self-reported through telephone survey)	Poor
Green et al., 2010	0<br>(those visiting a large urban public hospital on regular basis (US))	*	*<br>(administrative claims)	*	*	*<br>(administrative claims)	Fair
Chen et al., 2010	0<br>(assessed the effect of a pay-for-performance in a preferred provider organization, specific geographical area (Hawaii, US))	*	*<br>(administrative claims)	*	*	*<br>(administrative claims)	Fair
Gulliford et al., 2010	0<br>(specific geographical area (South London boroughs, England), deprived area)	*	*<br>(administrative claims)	*	*	*<br>(administrative claims)	Fair
Tomio et al., 2010	0<br>(two communities in south-western Japan, attending at a regular basis, national health insurance enrolees)	*	*<br>(administrative claims)	*	*	*<br>(administrative claims)	Fair
Wilf-Miron et al., 2010	*/0<br>(Maccabi Healthcare Services enrolees)	*	*<br>(administrative data)	*	*	*<br>(administrative data)	Fair
Gregg et al., 2010	*/0<br>(those enrolled in enrolled in one of 10 US managed care plans, type 2 diabetes)	*	*<br>(self-reported)	*	*	*<br>(self-reported and medical records)	Fair
Ng et al., 2010	*/0<br>(population-based (MEPS, US), aged 45+)	*	*<br>(self-reported through computer assisted survey)	*	*	0<br>(self-reported through computer assisted survey)	Poor
Wang et al., 2010	0<br>(those visiting 1 of 3 hospitals/clinics included in a given time period, relatively small sample size (China))	*	*<br>(self-reported through interview)	*	*	*<br>(medical chart and otherwise self-reported)	Fair
Lawrenson et al., 2009	0<br>(three general practices in Hamilton (New Zealand which may not be directly generalizable, type 2 diabetes)	*	*<br>(primary care medical records)	*<br>(age)	0	*<br>(primary care medical records)	Fair
Guthrie et al., 2009	*/0<br>(population-based, specific geographical area (Tayside, Scotland), type 2 diabetes)	*	*<br>(diabetes register)	*	*	*<br>(diabetes register)	Fair
Gnavi et al., 2009	0<br>(specific geographical area (Torino, Italy), not assumed to be generalizable because of the urban area and easy access to care)	*	*<br>(administrative claims data)	*	*	*<br>(administrative claims data)	Fair
Kirkbride et al., 2009	0<br>(Oregon's Medicaid program enrolees (US), working age adults)	*	*<br>(administrative data)	*	*	*<br>(administrative data)	Fair
Greenan et al., 2019	0<br>(those referred to a specific Diabetic Retinopathy Treatment Centre from the Irish National Diabetic Retinal Screening Programme)	*	*<br>(medical records)	*	*	*<br>(medical records)	Fair

The categories assessed included: (1) selection, (2) comparability, and (3) outcome. Good quality was defined as three stars (*) in the selection domain, one or two starts in the comparability domain, and one star in the outcome domain. Fair quality was defined as two starts in the selection domein, one or two stars in the comparability domain and one star in the outcome domain. Poor quality was defined as one or zero stars in the selection domein, zero stars in the comparability domain, and zero starts in the outcome domain.

References supplemental data

1. Swietek, K. E., Gaynes, B. N., Jackson, G. L., Weinberger, M. & Domino, M. E. Effect of the Patient-Centered Medical Home on Racial Disparities in Quality of Care. *J. Gen. Intern. Med.* **35**, 2304–2313 (2020).

2. Comer-HaGans, D. L., Austin, S., Ramamonjiarivelo, Z. & Matthews, A. K. Diabetes Standard of Care Among Individuals Who Have Diabetes With and Without Cognitive Limitation Disabilities. *Diabetes Educ.* **46**, 94–107 (2020).

3. Lu, Z. *et al.* Disparities in diabetes management among medicaid recipients with intellectual and developmental disabilities (IDD): Evidence from five U.S. states. *Disabil. Health J.* **13**, 100880 (2020).

4. Wei, W. *et al.* Exploring geographic variation of and influencing factors for utilization of four diabetes management measures in Swiss population using claims data. *BMJ Open Diabetes Res. Care* **8**, 1–11 (2020).

5. Youn, H. M., Lee, D. W. & Park, E. C. Association between community outpatient clinic care accessibility and the uptake of diabetic retinopathy screening: A multi-level analysis. *Prim. Care Diabetes* 2–7 (2020) doi:10.1016/j.pcd.2020.02.010.

6. Tan, X., Lee, L. K., Huynh, S., Pawaskar, M. & Rajpathak, S. Sociodemographic disparities in the management of type 2 diabetes in the United States. *Curr. Med. Res. Opin.* **36**, 967–976 (2020).

7. Meier, R., Valeri, F., Senn, O., Rosemann, T. & Chmiel, C. Quality performance and associated factors in Swiss diabetes care – A cross-sectional study. *PLoS One* **15**, 1–14 (2020).

8. Hirst, J. A., Farmer, A. J., Smith, M. C. & Stevens, R. J. Timings for HbA 1c testing in people with diabetes are associated with incentive payments: an analysis of UK primary care data. *Diabet. Med.* **36**, 36–43 (2019).

9. Bakke *et al.* Population, general practitioner and practice characteristics are associated with screening procedures for microvascular complications in Type 2 diabetes care in Norway. *Diabet. Med.* **36**, 1431–1443 (2019).

10. Dallo, F. J. *et al.* Diabetes Management Among Arab Americans Who Sought Care at a Large Metropolitan Hospital System in Michigan. *J. Immigr. Minor. Heal.* **21**, 490–496 (2019).

11. de Jong, M. *et al.* Sex differences in cardiovascular risk management for people with diabetes in primary care: A cross-sectional study. *BJGP Open* **3**, 1–11 (2019).

12. Whyte, M. B. *et al.* Disparities in glycaemic control, monitoring, and treatment of type 2 diabetes in England: A retrospective cohort analysis. *PLoS Med.* **16**, 1–18 (2019).

13. Du, Y. *et al.* Gender differences in cardiovascular risk profiles and diabetes care among adults with type 2 diabetes in Germany. (2019) doi:doi.org/10.1016/j.diabet.2018.05.011.

14. Kovács, N. *et al.* Factors Associated with Practice-Level Performance Indicators in Primary Health Care in Hungary: A Nationwide Cross-Sectional Study. *Int. J. Environ. Res. Public Health* **16**, 3153 (2019).

15. Greenan, E., Salim, M., Coakley, D. N. & James, M. The effect of geodemographic factors on the attendance rates at a regional diabetic retinopathy treatment centre. *Ir. J. Med. Sci.* **188**, 1207–1212 (2019).

16. Kamat, S., Gousse, Y., Muzumdar, J. & Gu, A. Trends and Disparities in Quality of Diabetes Care in the US: The National Health and Nutrition Examination Survey, 1999-2016. *Inov. Pharm.* **10**, 17 (2019).

17. An, J. J., Niu, F., Turpcu, A., Rajput, Y. & Cheetham, T. C. Adherence to the American Diabetes Association retinal screening guidelines for population with diabetes in the United States. *Ophthalmic*

Epidemiol. **25**, 257–265 (2018).

18. Ibáñez, B. *et al.* Socioeconomic inequalities in cardiometabolic control in patients with type 2 diabetes. *BMC Public Health* **18**, 408 (2018).

19. Bird, C. E. *et al.* Mapping the Gaps: Gender Differences in Preventive Cardiovascular Care among Managed Care Members in Four Metropolitan Areas. *Womens. Health Issues* **28**, 446–455 (2018).

20. Kreft, D., McGuinness, M. B., Doblhammer, G. & Finger, R. P. Diabetic retinopathy screening in incident diabetes mellitus type 2 in Germany between 2004 and 2013 - A prospective cohort study based on health claims data. *PLoS One* **13**, e0195426 (2018).

21. Kawamura, T., Sato, I., Tamura, H., Nakao, Y. M. & Kawakami, K. Influence of comorbidities on the implementation of the fundus examination in patients with newly diagnosed type 2 diabetes. *Jpn. J. Ophthalmol.* **62**, 68–76 (2018).

22. National Diabetes Audit - NHS Digital. https://digital.nhs.uk/data-and-information/publications/statistical/national-diabetes-audit.

23. Foreman, J. *et al.* Adherence to diabetic eye examination guidelines in Australia: The national eye health survey. *Med. J. Aust.* **206**, 402–406 (2017).

24. Mwangi, N. *et al.* Predictors of uptake of eye examination in people living with diabetes mellitus in three counties of Kenya. *Trop. Med. Health* **45**, 1–10 (2017).

25. LeBlanc, E. *et al.* Influence of a Pay-for-Performance Program on Glycemic Control in Patients Living with Diabetes by Family Physicians in a Canadian Province. *Can. J. Diabetes* **41**, 190–196 (2017).

26. Yoo, K.-H. *et al.* Regional variations in frequency of glycosylated hemoglobin (HbA1c) monitoring in Korea: A multilevel analysis of nationwide data. *Diabetes Res. Clin. Pract.* **131**, 61–69 (2017).

27. Bennett, K. J., McDermott, S., Mann, J. R. & Hardin, J. Receipt of recommended services among patients with selected disabling conditions and diabetes. *Disabil. Health J.* **10**, 58–64 (2017).

28. Williams, J. S., Bishu, K. G., Germain, A. St. & Egede, L. E. Trends in sex differences in the receipt of quality of care indicators among adults with diabetes: United States 2002-2011. *BMC Endocr. Disord.* **17**, (2017).

29. Willis, T. A. *et al.* Variations in achievement of evidence-based, high-impact quality indicators in general practice: An observational study. *PLoS One* **12**, e0177949 (2017).

30. Murchison, A. P. *et al.* Non-adherence to eye care in people with diabetes. *BMJ Open Diabetes Res. Care* **5**, 1–10 (2017).

31. Moreton, R. B. R., Stratton, I. M., Chave, S. J., Lipinski, H. & Scanlon, P. H. Factors determining uptake of diabetic retinopathy screening in Oxfordshire. *Diabet. Med.* **34**, 993–999 (2017).

32. Tanaka, H., Tomio, J., Sugiyama, T. & Kobayashi, Y. Process quality of diabetes care under favorable access to healthcare: A 2-year longitudinal study using claims data in Japan. *BMJ Open Diabetes Res.*

Care **4**, (2016).

33. Rossaneis, M. A., Haddad, M. do C. F. L., Mathias, T. A. de F. & Marcon, S. S. Diferenças entre mulheres e homens diabéticos no autocuidado com os pés e estilo de vida. *Rev. Lat. Am. Enfermagem* **24**, (2016).

34. Tannenbaum, S. L. *et al.* Ocular screening adherence across hispanic/latino heritage groups with diabetes: Results from the ocular SOL ancillary to the miami site of the hispanic community health study/study of latinos (HCHS/SOL). *BMJ Open Diabetes Res. Care* **4**, (2016).

35. Mtuya, C. *et al.* Reasons for poor follow-up of diabetic retinopathy patients after screening in Tanzania: A cross-sectional study. *BMC Ophthalmol.* **16**, 1–7 (2016).

36. Hatef, E., Vanderver, B. G., Fagan, P., Albert, M. & Alexander, M. Annual diabetic eye examinations in a managed care Medicaid population. *Am. J. Manag. Care* **21**, e297–e302 (2015).

37. Baumeister, S. E. *et al.* Trends of barriers to eye care among adults with diagnosed diabetes in Germany, 1997-2012. *Nutr. Metab. Cardiovasc. Dis.* **25**, 906–915 (2015).

38. Sieng, S., Thinkamrop, B. & Hurst, C. Achievement of Processes of Care for Patients with Type 2 Diabetes in General Medical Clinics and Specialist Diabetes Clinics in Thailand. *Epidemiol. Open Access* **s2**, (2015).

39. Mounce, L. T. A. *et al.* Patient characteristics predicting failure to receive indicated care for type 2 diabetes. *Diabetes Res. Clin. Pract.* **107**, 247–258 (2015).

40. Liang, H., Kennedy, C., Manne, S., Lin, J. H.-L. & Dolin, P. Monitoring for proteinuria in patients with type 2 diabetes mellitus. *BMJ Open Diabetes Res. Care* **3**, e000071 (2015).

41. Hwang, J., Rudnisky, C., Bowen, S. & Johnson, J. A. Socioeconomic factors associated with visual impairment and ophthalmic care utilization in patients with type II diabetes. *Can. J. Ophthalmol.* **50**, 119–126 (2015).

42. Casanova, L., Roses, F., Carrier, H., Gentile, G. & Verger, P. Evolution of paraclinical monitoring between 2008 and 2011of treated type 2 diabetic patients. **26**, 205–212 (2015).

43. Devkota, B. P., Ansstas, M., Scherrer, J. F., Salas, J. & Budhathoki, C. Internal Medicine Resident Training and Provision of Diabetes Quality of Care Indicators. *Can. J. Diabetes* **39**, 133–137 (2015).

44. Billimek, J. *et al.* Understanding disparities in lipid management among patients with type 2 diabetes: gender differences in medication nonadherence after treatment intensification. *Womens. Health Issues* **25**, 6–12 (2015).

45. Al Sayah, F., Soprovich, A., Qiu, W., Edwards, A. L. & Johnson, J. A. Diabetic Foot Disease, Self-Care and Clinical Monitoring in Adults with Type 2 Diabetes: The Alberta's Caring for Diabetes (ABCD) Cohort Study. *Can. J. Diabetes* **39**, S120–S126 (2015).

46. Doorn-Klomberg, A. L. Van *et al.* Patient Characteristics Associated with Measurement of Routine Diabetes Care: An Observational Study. *PLoS One* **10**, (2015).

47. Lee, D. J. *et al.* Dilated eye examination screening guideline compliance among patients with diabetes without a diabetic retinopathy diagnosis: the role of geographic access. *BMJ Open Diabetes Res. Care* **2**, e000031 (2014).

48. Maclennan, P. A. *et al.* Eye Care Utilization among a High-Risk Diabetic Population Seen in a Public Hospital's Clinics. *JAMA Ophthalmol.* **132**, 162–167 (2014).

49. Buja, A. *et al.* Need and disparities in primary care management of patients with diabetes. *BMC Endocr. Disord.* **14**, 1–8 (2014).

50. Naicker, K., Liddy, C., Singh, J., Taljaard, M. & Hogg, W. Quality of cardiovascular disease care in Ontario's primary care practices: a cross sectional study examining differences in guideline adherence by patient sex. *BMC Fam. Pract.* **15**, 123 (2014).

51. Baviera, M. *et al.* Sex differences in cardiovascular outcomes, pharmacological treatments and indicators of care in patients with newly diagnosed diabetes: Analyses on administrative database. *Eur. J. Intern. Med.* **25**, 270–5 (2014).

52. Chen, R., Cheadle, A., Johnson, D. & Duran, B. US Trends in Receipt of Appropriate Diabetes Clinical and Self-care From 2001 to 2010 and Racial/Ethnic Disparities in Care. *Diabetes Educ.* **40**, 756–766 (2014).

53. Rim, T. H. T., Byun, I. H., Kim, H. S., Lee, S. Y. & Yoon, J. S. Factors associated with diabetic retinopathy and nephropathy screening in Korea: The third and fourth Korea national health and nutrition examination survey (KNHANES III and IV). *J. Korean Med. Sci.* **28**, 814–820 (2013).

54. Yu, M. K., Lyles, C. R., Bent-Shaw, L. A. & Young, B. A. Sex disparities in diabetes process of care measures and self-care in high-risk patients. *J. Diabetes Res.* **2013**, (2013).

55. Rossi, M. C. *et al.* Sex disparities in the quality of diabetes care: Biological and cultural factors may play a different role for different outcomes: A cross-sectional observational study from the amd annals initiative. *Diabetes Care* **36**, 3162–3168 (2013).

56. Hellemons, M. E., Denig, P., De Zeeuw, D., Voorham, J. & Lambers Heerspink, H. J. Is albuminuria screening and treatment optimal in patients with type 2 diabetes in primary care? Observational data of the GIANTT cohort. *Nephrol. Dial. Transplant.* **28**, 706–715 (2013).

57. Mier, N. *et al.* Factors influencing health care utilization in older Hispanics with diabetes along the Texas-Mexico border. *Popul. Health Manag.* **15**, 149–156 (2012).

58. Druss, B. G. *et al.* Mental comorbidity and quality of diabetes care under Medicaid: a 50-state analysis. *Med. Care* **50**, 428–33 (2012).

59. Bartels, C. M. *et al.* Monitoring diabetes in patients with and without rheumatoid arthritis: a Medicare study. *Arthritis Res. Ther.* **14**, 1–9 (2012).

60. Chien, A. T., Eastman, D., Li, Z. & Rosenthal, M. B. Impact of a pay for performance program to improve diabetes care in the safety net. *Prev. Med. (Baltim).* **55**, S80–S85 (2012).

61. Kiran, T., Victor, J. C., Kopp, A., Shah, B. R. & Glazier, R. H. The relationship between financial incentives and quality of diabetes care in Ontario, Canada. *Diabetes Care* **35**, 1038–1046 (2012).

62. Reichard, A., Stolzle, H., Sella, A. C. & Shireman, T. I. Quality of diabetes care for adults with physical disabilities in Kansas. *Disabil. Health J.* **5**, 34–40 (2012).

63. Gold, R. *et al.* Receipt of diabetes preventive care among safety net patients associated with differing levels of insurance coverage. *J Am Board Fam Med* **25**, 42–49 (2012).

64. Kilbourne, A. M. *et al.* Quality of general medical care among patients with serious mental illness: Does colocation of services matter? *Psychiatr. Serv.* **62**, 922–928 (2011).

65. Stefos, T. *et al.* The effect of physician panel size on health care outcomes. *Heal. Serv. Manag. Res.* **24**, 96–105 (2011).

66. Fraser, S. *et al.* Sociodemographic differences in diabetic retinopathy screening; using patient-level primary care data for health equity audit. *Clin. Audit* 7 (2011) doi:10.2147/ca.s25313.

67. Williams, S. L. *et al.* Serious psychological distress and diabetes care among California adults. *Int. J. Psychiatry Med.* **40**, 233–245 (2010).

68. Green, J. L., Gazmararian, J. A., Rask, K. J. & Druss, B. G. Quality of diabetes care for underserved patients with and without mental illness: Site of care matters. *Psychiatr. Serv.* **61**, 1204–1210 (2010).

69. Chen, J. Y. *et al.* The effect of a PPO pay-for-performance program on patients with diabetes. *Am. J. Manag. Care* **16**, 11–19 (2010).

70. Tomio, J., Toyokawa, S., Tanihara, S., Inoue, K. & Kobayashi, Y. Quality of care for diabetes patients using National Health Insurance claims data in Japan. *J. Eval. Clin. Pract.* **16**, 1164–1169 (2010).

71. Wilf-Miron, R. *et al.* Disparities in diabetes care: role of the patient's socio-demographic characteristics. *BMC Public Health* **10**, 729 (2010).

72. Gregg, E. W. *et al.* Characteristics of Insured Patients with Persistent Gaps in Diabetes Care Services: The Translating Research into Action for Diabetes (TRIAD) Study. *Med Care* **48**, 31–37 (2010).

73. Ng, J. & Scholle, S. H. Disparities in Quality of Care for Midlife Adults (Ages 45-64) Versus Older Adults (Ages >65). 1–72 (2010).

74. Wang, D. *et al.* Use of eye care services among diabetic patients in Urban and Rural China. *Ophthalmology* **117**, 1755–1762 (2010).

75. Gulliford, M. *et al.* Socioeconomic and Ethnic Inequalities in Diabetes Retinal Screening. *Diabet. Med.* **27**, 282–8 (2010).

76. Lawrenson, R., Gibbons, V., Joshy, G. & Choi, P. Are there disparities in care in people with diabetes? A review of care provided in general practice. *J. Prim. Health Care* **1**, 177–83 (2009).

77. Guthrie, B., Emslie-Smith, A. & Morris, A. D. Which people with Type 2 diabetes achieve good control

of intermediate outcomes? Population database study in a UK region. *Diabet. Med.* **26**, 1269–1276 (2009).

78. Gnavi, R., Picariello, R., La Karaghiosoff, L., Costa, G. & Giorda, C. Determinants of quality in diabetes care process: The population-based Torino study. *Diabetes Care* **32**, 1986–1992 (2009).

79. Kirkbride, K. & Wallace, N. Rural health clinics and diabetes-related primary care for medicaid beneficiaries in oregon. *J. Rural Heal.* **25**, 247–252 (2009).

80. Peraj, E., Subhani, M. R., Jeong, J., Vaknin, O. S. & Twarog, J. P. Characteristics among adult patients with diabetes who received a foot exam by a health care provider in the past year: An analysis of NHANES 2011–2016. *Prim. Care Diabetes* **13**, 242–246 (2019).

81. Barker, L. C., Kurdyak, P., Jacob, B. & Vigod, S. N. Quality of Diabetes Care for Individuals with Comorbid Chronic Psychotic Illness: A Sex-Based Analysis. *J. Women's Heal.* **27**, 290–296 (2018).

82. Canedo, J. R., Miller, S. T., Schlundt, D., Fadden, M. K. & Sanderson, M. Racial/Ethnic Disparities in Diabetes Quality of Care: the Role of Healthcare Access and Socioeconomic Status. *J. Racial Ethn. Heal. Disparities* **5**, 7–14 (2018).

83. Sieng, S. & Hurst, C. A combination of process of care and clinical target among type 2 diabetes mellitus patients in general medical clinics and specialist diabetes clinics at hospital levels. *BMC Health Serv. Res.* **17**, (2017).

84. Doucette, E. D., Salas, J., Wang, J. & Scherrer, J. F. Insurance coverage and diabetes quality indicators among patients with diabetes in the US general population. *Prim. Care Diabetes* **11**, 515–521 (2017).

85. Storey, P. P. *et al.* Impact of physician communication on diabetic eye examination adherence: Results from a retrospective cohort analysis. *Retina* **36**, 20–27 (2016).

86. Sohn, M.-W. *et al.* Disparities in recommended preventive care usage among persons living with diabetes in the Appalachian region. *BMJ Open Diabetes Res. Care* **4**, e000284 (2016).

87. Mahmoudi, E., Tarraf, W., Maroukis, B. L. & Levy, H. G. Does Medicare Managed care reduce racial/ethnic disparities in diabetes preventive care and healthcare expenditures? *Am. J. Manag. Care* **22**, e360–e367 (2016).

88. Doucette, E. D., Salas, J. & Scherrer, J. F. Insurance Coverage and Diabetes Quality Indicators Among Patients in NHANES. *Am J Manag Care.* **22**, 484–490 (2016).

89. Shi, Q., Zhao, Y., Fonseca, V., Krousel-Wood, M. & Shi, L. Racial disparity of eye examinations among the U.S. working-age population with diabetes: 2002-2009. *Diabetes Care* **37**, 1321–1328 (2014).

90. Hu, R., Shi, L., Rane, S., Zhu, J. & Chen, C.-C. Insurance, racial/ethnic, SES-related disparities in quality of care among US adults with diabetes. *J. Immigr. Minor. Heal.* **16**, 565–75 (2014).

91. Chou, C. F. *et al.* Impact of geographic density of eye care professionals on eye care among adults with diabetes. *Ophthalmic Epidemiol.* **19**, 340–349 (2012).

92. Hale, N. L., Bennett, K. J. & Probst, J. C. Diabetes care and outcomes: Disparities across rural America. *J. Community Health* **35**, 365–374 (2010).

93. Byun, S. H., Ma, S. H., Jun, J. K., Jung, K. W. & Park, B. Screening for Diabetic Retinopathy and Nephropathy in Patients with Diabetes: A Nationwide Survey in Korea. *PLoS One* **8**, 1–8 (2013).

94. Richard, P., Alexandre, P. K., Younis, M. Z., Lara, A. & Akamigbo, A. B. Racial and Ethnic Disparities in the Quality of Diabetes Care for the Elderly in a Nationally Representative Sample. *Ageing Int.* **37**, 155–164 (2012).

95. Richard, P., Alexandre, P. K., Lara, A. & Akamigbo, A. B. Racial and Ethnic Disparities in the Quality of Diabetes Care in a Nationally Representative Sample. *Prev Chronic Dis* **8**, A142 (2011).

96. Do, Y. K. & Eggleston, K. N. Educational disparities in quality of diabetes care in a universal health insurance system: Evidence from the 2005 Korea National Health and Nutrition Examination Survey. *Int. J. Qual. Heal. Care* **23**, 397–404 (2011).

97. Backe, M. B. & Pedersen, M. L. Prevalence, incidence, mortality, and quality of care of diagnosed diabetes in Greenland. *Diabetes Res. Clin. Pract.* **160**, 107991 (2020).

98. Boucher, M. C., Ouazani Chahdi, H. & El Yamani, M. E. M. Compliance to follow-up care after urban diabetic retinopathy tele-screening. *Can. J. Ophthalmol.* **55**, 2–7 (2020).

99. Benoit, S. R., Swenor, B., Geiss, L. S., Gregg, E. W. & Saaddine, J. B. Eye Care Utilization Among Insured People With Diabetes in the U.S., 2010–2014. *Diabetes Care* **42**, 427–433 (2019).

100. Gediminas, R., Ida, L., Lina, J. & Valius, L. Guideline Adherence and the Factors Associated with Better Care for Type 2 Diabetes Mellitus Patients in Lithuanian PHC: Diabetes Mellitus Guideline Adherence in Lithuania PHC. *Open Med. J.* **6**, 50–57 (2019).

101. Wright, A. K. *et al.* Cardiovascular Risk and Risk Factor Management in Type 2 Diabetes Mellitus: A Population-Based Cohort Study Assessing Sex Disparities. *Circulation* **139**, 2742–2753 (2019).

102. Nazu, N. A. *et al.* Maintenance of good glycaemic control is challenging - A cohort study of type 2 diabetes patient in North Karelia, Finland. *Int. J. Clin. Pract.* **73**, e13313 (2019).

103. Corrao, G. *et al.* Effectiveness of adherence to recommended clinical examinations of diabetic patients in preventing diabetes-related hospitalizations. *Int. J. Qual. Heal. Care* **31**, 464–472 (2019).

104. Tracey, M., Racine, E., Riordan, F., McHugh, S. M. & Kearney, P. M. Understanding the uptake of a national retinopathy screening programme: An audit of people with diabetes in two large primary care centres. *HRB Open Res.* **2**, 17 (2019).

105. Mesa, M. S. Health care disparities between men and women with type 2 diabetes. *Prev. Chronic Dis.* **15**, 1–6 (2018).

106. Al-Salameh, A. *et al.* Gender-Related Differences in the Control of Cardiovascular Risk Factors in Primary Care for Elderly Patients With Type 2 Diabetes: A Cohort Study. *Can. J. diabetes* **42**, 365-371.e2 (2018).

107. Bird, C. E. *et al.* How Do Gender Differences in Quality of Care Vary Across Medicare Advantage Plans? *J. Gen. Intern. Med.* **33**, 1752 (2018).

108. Diabetic RetinaScreen Programme Report 2013-2015.

109. Diabetic RetinaScreen Statistical Bulletin 2016-2017. (2017).

110. Kekäläinen, P., Tirkkonen, H. & Laatikainen, T. How are metabolic control targets of patients with Type 1 diabetes mellitus achieved in daily practice in the area with high diabetes prevalence? *Diabetes Res. Clin. Pract.* **115**, 9–16 (2016).

111. Han, J. A., Kim, S. J., Kim, G., Kim, E. J. & Lee, S. Y. Factors affecting screening for diabetic complications in the community: a multilevel analysis. *Epidemiol. Health* **38**, e2016017 (2016).

112. Ferroni, E. *et al.* Patient and General Practitioner characteristics influencing the management of non-insulin-treated diabetes mellitus: A cross-sectional study in Italy. *Diabetes Res. Clin. Pract.* **116**, 192–201 (2016).

113. Cambra, K. *et al.* Sex and age differences in the achievement of control targets in patients with type 2 diabetes: results from a population-based study in a South European region. *BMC Fam. Pract.* **17**, 1–7 (2016).

114. Seghieri, C., Policardo, L., Francesconi, P. & Seghieri, G. Gender differences in the relationship between diabetes process of care indicators and cardiovascular outcomes. *Eur. J. Public Health* **26**, 219–224 (2016).

115. Cleland, C. R. *et al.* Diabetic retinopathy in Tanzania: prevalence and risk factors at entry into a regional screening programme. *Trop. Med. Int. Heal.* **21**, 417–426 (2016).

116. Manicardi, V. *et al.* Gender-Disparities in Adults with Type 1 Diabetes: More Than a Quality of Care Issue. A Cross-Sectional Observational Study from the AMD Annals Initiative. *PLoS One* **11**, e0162960 (2016).

117. Hwang, J. Decomposing socioeconomic inequalities in the use of preventive eye screening services among individuals with diabetes in Korea. *Int. J. Public Health* **61**, 613–620 (2016).

118. Keenum, Z. *et al.* Patients' adherence to recommended follow-up eye care after diabetic retinopathy screening in a publicly funded county clinic and factors associated with follow-up eye care use. *JAMA Ophthalmol.* **134**, 1221–1228 (2016).

119. Szabo, S. M. *et al.* Quality of Care for Patients with Type 2 Diabetes Mellitus in Dubai: A HEDIS-Like Assessment. *Int. J. Endocrinol.* **2015**, 1–8 (2015).

120. Afandi, B., Malik, A. A., AlKaabi, J., Elhouni, A. & Aziz, F. Clinical Diabetes Care of Patients with Type 2 Diabetes at a Major Tertiary Care Hospital in the United Arab Emirates. *J. Diabetes, Metab. Disord. Control* **2**, 7–12 (2015).

121. Hendriks, S. H. *et al.* Sex Differences in the Quality of Diabetes Care in the Netherlands (ZODIAC-45). *PLoS One* **10**, e0145907 (2015).

122. Ballotari, P. *et al.* Differences in diabetes prevalence and inequalities in disease management and glycaemic control by immigrant status: a population-based study (Italy). *BMC Public Health* **15**, 87 (2015).

123. Russo, G. *et al.* Age- and Gender-Related Differences in LDL-Cholesterol Management in Outpatients with Type 2 Diabetes Mellitus. *Int. J. Endocrinol.* **2015**, 957105 (2015).

124. Onakpoya, O. H., Kolawole, B. A., Adeoye, A. O. & Okunoye, O. A. Compliance with diabetic retinopathy screening in a Nigerian tertiary hospital. *African J. Diabetes Med.* **23**, 20–22 (2015).

125. Kiran, T., Victor, J. C., Kopp, A., Shah, B. R. & Glazier, R. H. The Relationship between primary care models and processes of diabetes care in Ontario. *Can. J. Diabetes* **38**, 172–178 (2014).

126. Bayer, F. J. *et al.* complications. **20**, 41–52 (2014).

127. Chou, C.-F. *et al.* Barriers to Eye Care Among People Aged 40 Years and Older With Diagnosed Diabetes, 2006–2010. *Diabetes Care* **37**, 180–188 (2014).

128. Matheka, D. M., Kilonzo, J. M., Munguti, C. M. & Mwangi, P. W. Pattern, knowledge and practices of HbA1C testing among diabetic patients in a Kenyan tertiary referral hospital. *Global. Health* **9**, 1 (2013).

129. Kiran, T. *et al.* Unintended consequences of delisting routine eye exams on retinopathy screening for people with diabetes in Ontario, Canada. *Cmaj* **185**, 167–173 (2013).

130. Çetin, E. N., Zencir, M., Fenkçi, S., Akin, F. & Yildirim, C. Assessment of awareness of diabetic retinopathy and utilization of eye care services among Turkish diabetic patients. *Prim. Care Diabetes* **7**, 297–302 (2013).

131. Paksin-Hall, A., Dent, M. L., Dong, F. & Ablah, E. Factors contributing to diabetes patients not receiving annual dilated eye examinations. *Ophthalmic Epidemiol.* **20**, 281–287 (2013).

132. Driskell, O. J. *et al.* Inappropriate requesting of glycated hemoglobin (Hb A1c) is widespread: Assessment of prevalence, impact of national guidance, and practice-To-practice variability. *Clin. Chem.* **58**, 906–915 (2012).

133. Orton, E., Forbes-Haley, A., Tunbridge, L. & Cohen, S. Equity of uptake of a diabetic retinopathy screening programme in a geographically and socio-economically diverse population. *Public Health* **127**, 814–821 (2013).

134. Sachdeva, A., Stratton, I., Unwin, J., Moreton, R. & Scanlon, P. Diabetic retinopathy screening: Study to determine risk factors for non-attendance. *Diabetes Prim. Care* **14**, 308–316 (2012).

135. Arcury, T. A. *et al.* Social Integration and Diabetes Management among Rural Older Adults. *J. Aging Health* **24**, 899–922 (2012).

136. Van Eijk, K. N. D., Blom, J. W., Gussekloo, J., Polak, B. C. P. & Groeneveld, Y. Diabetic retinopathy screening in patients with diabetes mellitus in primary care: Incentives and barriers to screening attendance. *Diabetes Res. Clin. Pract.* **96**, 10–16 (2012).

137. Wong, K. W., Ho, S. Y. & Chao, D. V. K. Quality of diabetes care in public primary care clinics in Hong Kong. *Fam. Pract.* **29**, 196–202 (2012).

138. Sundquist, K., Chaikiat, A., Leon, V. I., Johansson, S.-E. & Sundquist, J. Country of birth, socioeconomic factors, and risk factor control in patients with type 2 diabetes: a Swedish study from 25 primary health-care centres. *Diabetes/Metabolism Research and Reviews* 244–254 (2011) doi:10.1002/dmrr.1161.

139. Sadowski, D., Devlin, M. & Hussain, A. Better care at safety net providers? Utilization of recommended standards of diabetes care for rural Latinos in one Midwestern state. *J. Health Care Poor Underserved* **22**, 995–1013 (2011).

140. De lusignan, S. *et al.* Disparities in testing for renal function in UK primary care: Cross-sectional study. *Fam. Pract.* **28**, 638–646 (2011).

141. Morren, J. A., Baboolal, N., Davis, G. K. & McRae, A. Assessment of treatment goals attained by patients according to guidelines for diabetes management in primary care centres in North Trinidad. *Qual. Prim. Care* **18**, 335–343 (2010).

142. Onakpoya, O. H., Adeoye, A. O. & Kolawole, B. A. Determinants of previous dilated eye examination among type II diabetics in Southwestern Nigeria. *Eur. J. Intern. Med.* **21**, 176–179 (2010).

143. Goh, P., Omar, M. A. & Yusoff, A. F. Diabetic eye screening in Malaysia: Findings from the National Health and Morbidity Survey 2006. *Singapore Med. J.* **51**, 631–634 (2010).

144. Gossain, V. V., Rosenman, K. D., Gardiner, J. C., Thawani, H. T. & Tang, X. Evaluation of control of diabetes mellitus in a subspecialty clinic. *Endocr. Pract.* **16**, 178–186 (2010).

145. Shireman, T. I., Reichard, A., Nazir, N., Backes, J. M. & Greiner, K. A. Quality of diabetes care for adults with developmental disabilities. *Disabil. Health J.* **3**, 179–185 (2010).

146. Banta, J. E., Morrato, E. H., Lee, S. W. & Haviland, M. G. Retrospective analysis of diabetes care in california medicaid patients with mental illness. *J. Gen. Intern. Med.* **24**, 802–808 (2009).

147. Fischbacher, C. M., Bhopal, R., Steiner, M., Morris, A. D. & Chalmers, J. Is there equity of service delivery and intermediate outcomes in South Asians with type 2 diabetes? Analysis of DARTS database and summary of UK publications. *J. Public Health (Bangkok).* **31**, 239–249 (2009).
